# Novel compounds that synergize with aminoglycoside G418 or eRF3 degraders for translational readthrough of nonsense mutant *TP53* and *PTEN*

**DOI:** 10.1080/15476286.2023.2222250

**Published:** 2023-06-20

**Authors:** Angelos Heldin, Matko Cancer, Mireia Palomar-Siles, Susanne Öhlin, Meiqiongzi Zhang, Alexander Sun-Zhang, Anna Mariani, Jianping Liu, Vladimir J.N. Bykov, Klas G. Wiman

**Affiliations:** a Department of Oncology-Pathology, BioClinicum, Karolinska Institutet, Stockholm, Sweden; b Department of Medicine, Karolinska Institutet, Huddinge, Sweden

**Keywords:** *TP53*, *PTEN*, nonsense mutation, novel compounds, translational readthrough, synergy

## Abstract

The *TP53* and *PTEN* tumour suppressor genes are inactivated by nonsense mutations in a significant fraction of human tumours. *TP53* nonsense mutatant tumours account for approximately one million new cancer cases per year worldwide. We have screened chemical libraries with the aim of identifying compounds that induce translational readthrough and expression of full-length p53 protein in cells with nonsense mutation in this gene. Here we describe two novel compounds with readthrough activity, either alone or in combination with other known readthrough-promoting substances. Both compounds induced levels of full-length p53 in cells carrying R213X nonsense mutant *TP53*. Compound C47 showed synergy with the aminoglycoside antibiotic and known readthrough inducer G418, whereas compound C61 synergized with eukaryotic release factor 3 (eRF3) degraders CC−885 and CC−90009. C47 alone showed potent induction of full-length PTEN protein in cells with different *PTEN* nonsense mutations. These results may facilitate further development of novel targeted cancer therapy by pharmacological induction of translational readthrough.

## Introduction

The tumour protein 53 gene (*TP53)* codes for the tumour suppressor protein p53, a transcription factor that regulates a multitude of cellular processes including cell cycle progression, metabolism, DNA repair and cell death by apoptosis [1]. p53 is activated in response to cellular stress, for instance DNA damage, oncogenic signalling and/or metabolic stress (e.g. glucose deprivation, hypoxia), leading to cell growth inhibition and tumour suppression by a variety of mechanisms [2]. p53 regulates a core transcriptional programme that mediates the many functions of p53 [3]. Levels of p53 are kept low under normal conditions due to stringent regulation by the E3 ligase Mdm2 that ubiquitinates p53, tagging it for proteasomal degradation [4]. However, upon cellular stress, p53 is subjected to post-translational modifications that prevent Mdm2-p53 binding, causing increased p53 protein levels and activation of p53 target genes [5,6].

The phosphatase and tensin homolog (*PTEN*) gene was first identified as a putative tumour suppressor gene on human chromosome 10q23 [7–9]. It encodes a phosphatase that regulates the PI3K (phosphatidylinositol−3 kinase)-AKT (Protein kinase B; PKB) pathway [10]. Its main substrate is phosphatidylinositol−3, 4, 5-triphosphate (PIP_3_). Accumulated PIP_3_ stimulates cell growth through the PI3K-AKT pathway by acting as a docking site for Akt kinase at the plasma membrane, allowing Akt activation by the PDK1/PDK2 kinases (3-Phosphoinositide-dependent kinase 1/2) and subsequent downstream signal transduction. PTEN inhibits the PI3K-AKT pathway by converting PIP_3_ to PIP_2_. The PI3K-AKT pathway regulates many important cellular processes and pathways, including cell growth and survival, cell size, metabolism and DNA repair [10,11].

Mutations that inactivate the tumour suppressor genes *TP53* and *PTEN* occur with high frequency in a wide range of tumour types [12]. According to the World Health Organization (WHO), over 19 million new cancer cases are reported annually worldwide [13]. Around 50% of all tumours carry *TP53* mutations [14]. While the majority of these mutations (70%) are missense mutations, 11% of *TP53* mutations are nonsense mutations, according to the COSMIC database (https://cancer.sanger.ac.uk/cosmic/gene/analysis?ln=TP53). Thus, approximately one million new cases of cancer carrying nonsense mutant *TP53* are diagnosed each year [15]. R213X is the most common TP53 nonsense mutation and the sixth most common TP53 mutation overall [16].

*PTEN* mutations have been detected in 63.5% of uterine corpus endometrial carcinomas and 30.7% of glioblastomas, and in 9.7% of the cases of 12 common tumour types overall [12]. As much as 18% of all inactivating *PTEN* mutations in cancer are nonsense mutations, of which the most common are R130X, R233X and R335X [15]. These mutations are located either in the phosphatase domain (R130X) or the lipid affinity domain (R233X and R335X) of the PTEN protein [11]. R233X is the most common *PTEN* nonsense mutation and the second most common *PTEN* mutation overall [15].

A nonsense mutation causes a premature termination codon (PTC) within the coding sequence of a gene. When the ribosome reaches a PTC in the mRNA, translational termination results in release of a truncated inactive protein. Translational termination is dependent on competition for binding to the stop codon between the eukaryotic release factor (eRF) 1 and near-cognate tRNAs [17]. At a very low frequency, a near-cognate tRNA can outcompete eRF1 for binding to the decoding centre of the ribosome, allowing translational readthrough by insertion of an amino acid to the growing polypeptide chain [18].

To date, there is no approved targeted treatment for nonsense mutant *TP53* or *PTEN* in cancer. Aminoglycoside antibiotics have been shown to induce translational readthrough in various genes with nonsense mutations, including *TP53*, *PTEN*, *APC* (adenomatous polyposis coli), *ATM* (ataxia telangiectasia mutated), *CFTR* (cystic fibrosis transmembrane conductance regulator), and *DMD* (Duchenne muscular dystrophy) [19–23]. However, treatment with the most potent aminoglycosides G418 and gentamicin is associated with severe nephrotoxicity [24] and ototoxicity [25], making them unsuitable for long-term cancer therapy. The readthrough effect of aminoglycosides in nonsense mutant *TP53* cells can be potentiated by combination with Mdm2 inhibitors Nutlin−3a and MI−773 as well as proteasome inhibitor Bortezomib [22], suggesting that combination therapy may allow less toxic doses of aminoglycosides. Moreover, attempts have been made to alter the structure of existing aminoglycosides to reduce toxicity while maintaining readthrough activity. The aminoglycoside derivative NB124 (ELX−02) is currently in phase 2 clinical trials in cystic fibrosis patients with nonsense mutations in the *CFTR* gene [26,27]. The novel readthrough-inducing compound PTC124 (Ataluren) has been tested in phase III clinical trials [28]. Despite inconsistent activity, it has been given conditional authorization for the treatment of Duchenne muscular dystrophy by the European Medicines Agency (EMA) [26,29,30]. We have also found that the chemotherapeutic drug 5-Fluorouracil can induce translational readthrough of nonsense mutant *TP53* through its metabolite 5-Fluorouridine [31].

Here, we describe two novel compounds identified by chemical library screening, C47 and C61. We have characterized both compounds for readthrough activity in tumour cells with nonsense mutant *TP53* or *PTEN* as single treatments as well as in combination with previously reported readthrough-inducing compounds G418, CC−885 and CC−90009. Our results suggest that novel combination treatments should be explored for therapeutic targeting of nonsense mutant *TP53* and *PTEN* in cancer.

## Materials and methods

### Chemicals

C47 (T5966960) and C61 (T0501–7450) were purchased from Enamine (Ukraine). G418 (CAS nr: 49863-47-0) was obtained from Gibco (USA) and Nutlin−3a (CAS nr: 675576-98-4) from Sigma-Aldrich/Merck (Germany). CC−885 (CAS nr: 1010100-07-8) and CC−90009 (CAS nr: 1860875-51-9) were bought from Cayman Chemical (USA).

### Cell lines and cell culture

HDQ-P1 human breast cancer cells (DSMZ, Braunschweig, Germany) carry a homozygous nonsense mutation at codon 213 (CGA to TGA; R213X) in the *TP53* gene [32]. HDQ-P1 cells were grown in DMEM low glucose medium (Hyclone, USA) supplemented with 10% foetal bovine serum (FBS) (Gibco, USA) and 2.5 µg/ml plasmocin (Invivogen, Toulouse, France). H1299p53R213X cells were generated from H1299 p53 null human lung adenocarcinoma cells (ATCC, USA) by stable transfection with a pCMV-puro-bam plasmid harbouring the p53R213X coding sequence. Several cell lines with similar plasmid constructs were generated in this manner and used throughout this study **(Suppl. Fig. S1A)**. Stably transfected H1299 cells were cultured in RPMI−1640 medium (Hyclone, USA) supplemented with 10% FBS. Puromycin was added at 1 µg/ml to the culture medium to maintain selective pressure during culturing. Puromycin-free medium was used during all treatments. HCT116 is a human colorectal cancer cell line. All HCT116 sublines were cultured in McCoy’s 5A medium (Sigma-Aldrich/Merck, Germany) supplemented with 10% FBS (Gibco, USA). U251MG human glioblastoma cells were a generous gift from Arne Östman (Karolinska Institutet, Stockholm, Sweden) and were maintained in DMEM low glucose medium (Sigma-Aldrich/Merck, Germany) supplemented with 10% FBS (Gibco, USA). HCT116sfGFP150-UGA/UAG/UAA cells were a generous gift from Simon Elsässer and Birthe Meineke (Karolinska Institutet, Stockholm, Sweden). A schematic representation of all plasmid constructs used is shown in **Suppl. Fig. S1A**.

### Plasmid and lentivirus preparation

pCMV-puro-bam PTEN-R130X-TGA-∆C-FLAG, pCMV-puro-bam PTEN-R233X-TGA-∆C-FLAG, and pCMV-puro-bam-PTEN R335X-TGA-∆C-FLAG plasmids were designed using SnapGene *in silico* by mutating Arg130 to TGA (Arg130stop), Arg233 to TGA (Arg233stop), or Arg335 to TGA (Arg335stop) in the *PTEN* coding sequence and replacing the C-terminal region 3’ of each introduced premature stop codon with a FLAG tag. Similarly, pLenti CMV Puro PTEN_R130X_FLAG_eGFP, pLenti CMV Puro PTEN_R233X_FLAG_eGFP and pLenti CMV Puro PTEN_R335X_FLAG_eGFP were designed using SnapGene *in silico* by mutating Arg130 to TGA (Arg130stop), Arg233 to TGA (Arg233stop) or Arg335 to TGA (Arg335stop) in the *PTEN* coding sequence and replacing the C-terminal region 3’ of each introduced premature stop codon with the FLAG-T2A-EGFP sequence [33]. pLenti CMV Puro DEST (w118–1) was acquired from AddGene [34]. Plasmid constructs were generated by GeneScript (USA). Plasmids were produced in DH5α bacteria and purified using PureLink HiPure Plasmid Maxiprep Kit according to manufacturer’s protocol. Lentiviruses were produced as described [33].

### High-throughput screening of chemical libraries

HDQ-P1 cells were plated in 384-well plates and treated with compounds from chemical libraries provided by Karolinska High-Throughput Center (Table 1). After 72 h incubation with individual compounds, cells were fixed, permeabilized and blocked before staining with anti-p53 antibody DO−1 (Santa Cruz, USA) as well as with Hoechst (Thermo Fisher Scientific, USA) for nuclei counter staining. Plates were scanned by Acumen® Cellista (TTP Labtech, UK) for background fluorescence. Cells were then incubated with secondary antibodies conjugated with Alexa Fluor 488 fluorochrome (Thermo Fisher Scientific, USA) and reanalysed by Acumen® Cellista. The p53 signal was calculated based on the signal from the secondary antibody minus the background signal.

**Table 1.** Chemical libraries used for high-throughput screening. The name and the total number of compounds in each library is indicated.

Libraries	Number of compounds
Enamine (Diverse library)	28200
Prestwick (Known drugs)	1200
NIH (Clinical collection)	730
SelleckChem (Pharmacologically active)	1000
Tocris (Tocrisscreen)	1100
BioMed and SelleckChem	80 + 190
Enzo (Selected libraries)	500
Total	33000

### Western blotting

H1299, U251 and HCT116-derived cell lines were seeded in 6-well plates at a density of 200 000 to 100 000 cells per well. After 24 h, cells were treated with substances to be tested at indicated concentrations. After 72 h incubation, H1299 and HCT116 cells were harvested and lysed with lysis buffer containing 100 mM Tris pH 7.4, 150 mM NaCl and 1% NP40. Protease Inhibitor Cocktail (Sigma-Aldrich/Merck, Germany) was added prior to use. The U251 cells were lysed in RIPA-buffer containing 150 mM NaCl 1%, NP40 0.5%, sodium deoxycholate 0.1% and SDS 50 mM Tris-Cl pH 8. The protein concentrations were determined using Bradford protein assay (Bio-Rad, USA) or DC ^TM^ Protein assay (Bio-Rad, USA). Between 25 and 60 µg of protein was loaded on 10% SDS/PAGE gels (Thermo Fisher Scientific, USA) and run in 1 × MOPS (Thermo Fisher Scientific, USA). Gels were blotted on nitrocellulose membranes using iBlot2 (Thermo Fisher Scientific, USA). Membranes were blocked with 5% skimmed milk in PBS-T and then incubated with antibodies against p53 (DO−1, Santa Cruz, USA), FLAG (M2, Sigma-Aldrich/Merck, Germany), GAPDH (0411, Santa Cruz, USA), GFP (B−2, Santa Cruz, USA), eRF3a/GSPT1 (Thermo Fisher Scientific, USA) or eRF1 (Thermo Fisher Scientific, USA). Membranes were soaked in SuperSignal™ West Femto Maximum Sensitivity Substrate (Thermo Fisher Scientific, USA) and detected by Luminescent Image Analyzer LAS−1000 plus (Fujifilm, Japan) or iBright FL1000 imaging system (Thermo Fisher Scientific, USA). Western blot quantification was performed using ImageJ analysis software [35]. Each p53 band was normalized to its corresponding GAPDH band and then normalized to the untreated sample on the same blot.

### WST−1 assay

U251R130X/R233X/R335X and U251EV cells were seeded at 5000 cells/well in 96-wells plates and treated with C47 and G418 at indicated concentrations. After 72 h, water-soluble Tetrazolium−1 dye (WST−1) (Roche, Switzerland) was added according to the manufacturer’s specifications and absorbance was read at 440 nm with Varioskan^TM^ LUX multimode microplate reader (Thermo Scientific, USA).

### Enzyme-linked immunosorbent assay (ELISA)

96-well plates (Thermo Fisher Scientific, USA) were coated with 100 µl of 5 µg/ml anti-FLAG antibody FLAG M2 (Sigma-Aldrich/Merck, Germany) in 0.1 M carbonate buffer pH 9.2 overnight at 4°C. 250 µl blocking buffer (5% milk in PBS-T) was added to each well and incubated for 2 h and then washed with PBS-T before use. 100 µg protein in 50 µl lysis buffer and 50 µl blocking buffer from each sample lysate was loaded onto the coated 96-well plates and incubated at 4°C overnight. Following washing with PBS-T, DO−1 antibody conjugated with horseradish peroxidase (HRP) (Santa Cruz, USA) was used diluted 1:500 in blocking buffer and 100 µl of the diluted antibody was added to each well. The plates were incubated 2 h at 4°C before washing with PBS-T. Finally, 100 µl of 1-Step ^TM^ Ultra TMB-ELISA Substrate Solution (Thermo Fisher Scientific, USA) was added. The enzymatic reaction was carried out at room temperature until an adequate signal was obtained. The reaction was stopped by adding of 10 µl 1 M HCL. Absorbance was measured at 450 nm on Varioskan™ LUX multimode microplate reader (Thermo Fisher Scientific, USA).

### Immunofluorescence staining

H1299p53R213X-FLAG and H1299p53R213X-EGFP cells were grown on coverslips and treated as indicated prior to fixation with 4% PFA and permeabilized with 0.2% Triton in PBS. H1299p53R213X-FLAG cells were blocked with 2% BSA in PBS for 1 h prior to incubation with anti-FLAG antibody conjugated with AF647 (Cell Signalling, USA) and then mounted using Fluoroshield^TM^ with DAPI (Sigma-Aldrich/Merck, Germany). The results were visualized using a Zeiss AxioImager M2 microscope.

### Flow cytometry

H1299p53R213X-EGFP and H1299p53R213X-FLAG cells were seeded onto 12-well plates at a cell density of 100 000 cells/well. After 24 h, cells were treated at indicated concentrations of C47 and G418. Following 72 h incubation, cells were harvested by trypsinization before analysing EGFP-expression by FACS using NovoCyte flow cytometer (ACEA Biosciences, USA). H1299p53R213X-FLAG cells were used to assess the background fluorescence from the compounds. Neither C47 nor G418 introduced any significant amount of background fluorescence. Regardless, any background fluorescence from the H1299p53R213X-FLAG cells was subtracted from the corresponding sample in the H1299p53R213X-EGFP cells.

### Statistics and assessment of synergy

Data were statistically analysed using GraphPad Prism version 9.5.0 (GraphPad Software, USA). As all analysed data were normally distributed according to the Shapiro–Wilk test and more than two groups were analysed, repeated measures one-way ANOVA (Analysis of Variance) was used followed by Tukey’s multiple comparisons to analyse the data. The data were considered statistically significant when p-values were lower than 0.05.

Synergy calculations were made using the online tool SynergyFinder 3.0 [36]. A four-parameter logistic regression (LL4) curve-fitting algorithm was used to fit single-drug dose response curves, and the Zero interaction potency (ZIP) model was used to assess synergy. Synergy scores less than −10 were considered antagonistic, those between −10 and 10 were considered additive and those larger than 10 were considered synergistic.

## Results

### Chemical library screening for readthrough-inducing compounds

We designed a high-throughput screening pipeline **(Suppl. Fig. S1B)** for identifying novel readthrough-inducing compounds using HDQ-P1 human breast cancer cells homozygous for R213X nonsense mutant *TP53*. Induction of translational readthrough leading to increased levels of p53 protein was detected by immunofluorescence staining with the N-terminal p53 antibody DO−1. In total, over 33 000 compounds were screened from different chemical libraries (Table 1) at 10 µM during the first round of screening (data not shown). The 440 best hit compounds were selected for a second round of screening at three concentrations, 0.5 µM, 2 µM and 10 µM (data not shown). In a third round of screening, 34 selected compounds were tested at 0.5 µM, 1 µM, 2.5 µM, 5 µM, 10 µM and 20 µM for their potential to induce expression of p53 (data not shown). These 34 candidate readthrough-inducing compounds were further validated by an ELISA assay based on DO−1 and FLAG antibodies and originally *TP53* null H1299p53R213X-∆C-FLAG cells stably transfected with a *TP53* cDNA construct with the coding sequence up to the premature termination codon R213X followed by a FLAG tag **(Suppl. Fig. S1C)**. The ELISA data confirmed that several of the compounds identified in the screening were able to induce p53 protein levels in H1299p53R213X-∆C-FLAG cells.

### Screening validation and identification of C47 and C61 as top candidates

**Figure 1.**
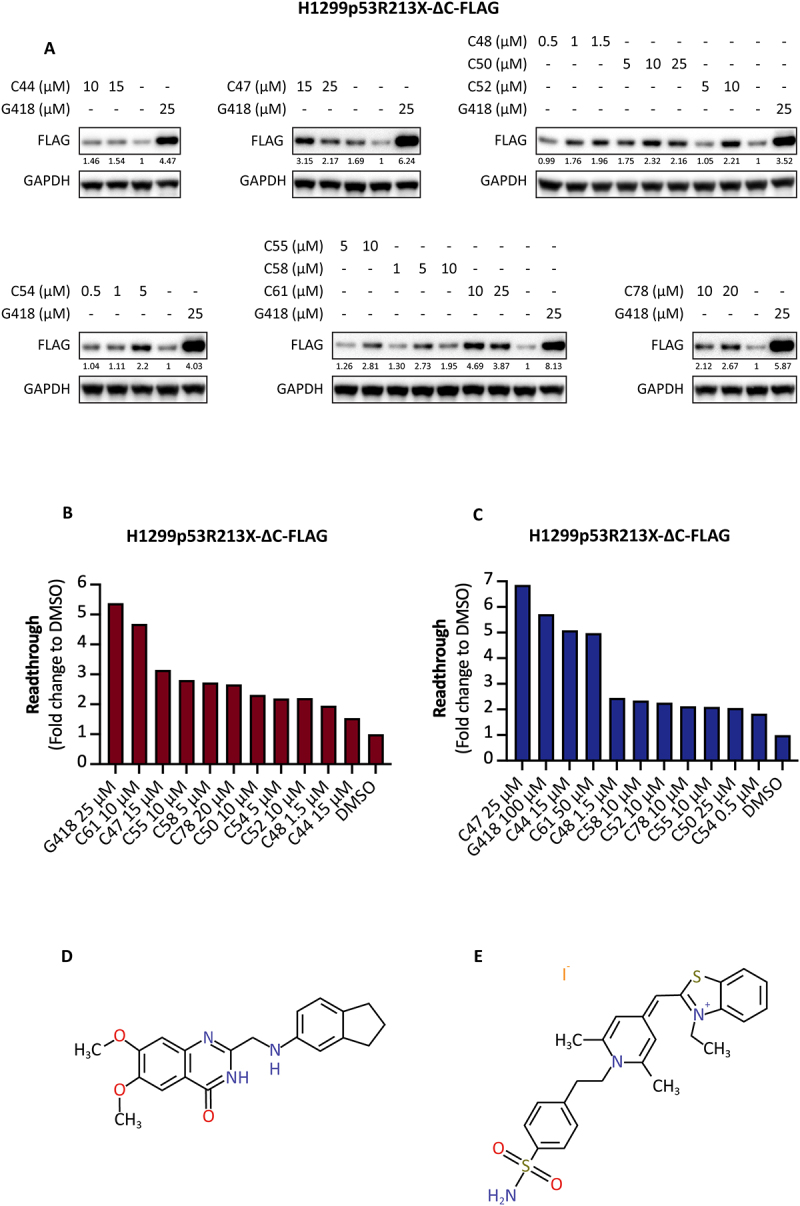
**Validation of high-throughput screening. A)** Western blot analysis of H1299p53R213X-ΔC-FLAG cells treated with top 10 candidate compounds from the high-throughput screening. Induction of readthrough was assessed using FLAG antibody. Quantification is shown underneath each band. GAPDH was used as loading control. **B)** Quantified Western blot data from fig. 1A presented as a bar chart. **C)** Readthrough induction by the top 10 candidate compounds H1299p53R213X-ΔC-FLAG cells as assessed by ELISA. **D, E)** Chemical structures of the selected top compounds C47 **(D)** and C61 **(E)**.

Ten compounds were selected based on the ELISA results and were further validated by Western blotting using H1299p53R213X-∆C-FLAG cells to assess induction of a truncated p53 protein with a C-terminal FLAG epitope. This analysis confirmed that several compounds induced expression of the p53-FLAG fusion protein, indicating translational readthrough (Figure 1A). Western blot quantification is presented in Figure 1B. These data were largely consistent with the ELISA data for the same set of compounds (Figure 1C). Based on the quantified Western and ELISA data, we selected the compounds C47 (2-[(2,3-dihydro−1 H-inden−5-ylamino)methyl]−6,7-dimethoxy−3 H-quinazolin−4-one) (Figure 1D) and C61 (4-[2-[4-[(3-Ethyl−1,3-benzothiazol−2-ylidene)methyl]−2,6-dimethylpyridin−1-ium−1-yl]ethyl]benzenesulfonamide;iodide) (Figure 1E) as the most promising candidates.

### Assessment of readthrough activity of C47 and C61

**Figure 2.**
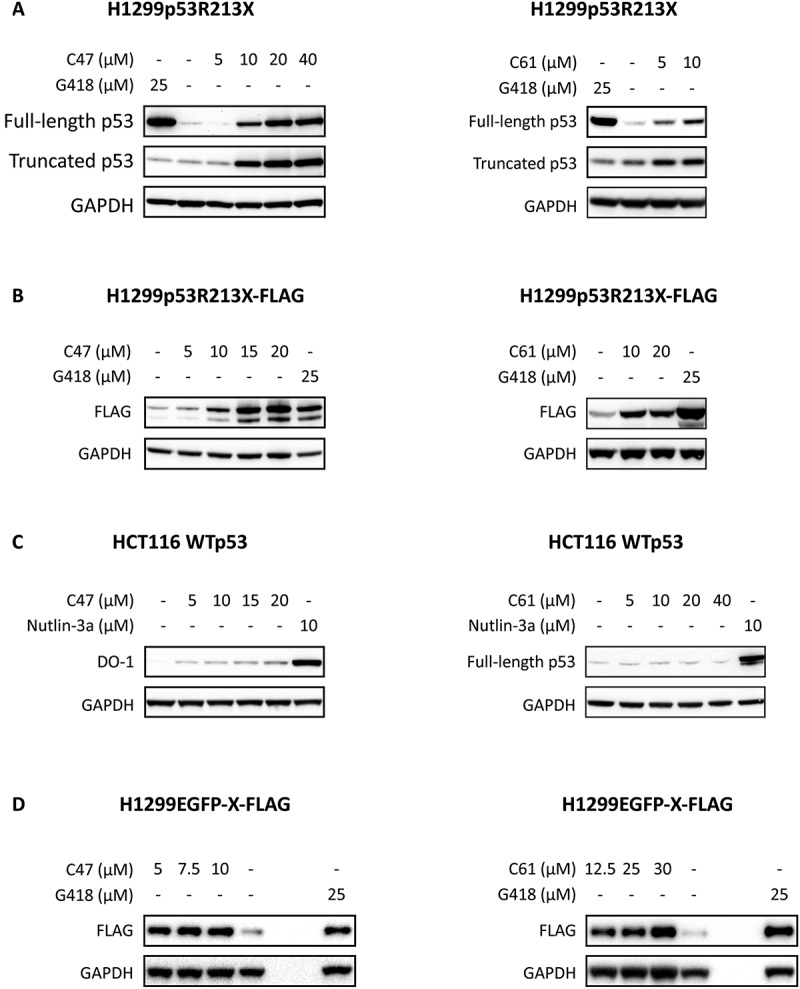
**Confirmation of readthrough activity of C47 and C61.** Induction of full-length p53 or FLAG by C47 and C61 was assessed by Western blotting in H1299p53R213X **(A)** and H1299p53R213X-FLAG **(B)** cells. **C)** Stabilization of full-length wild type (WT) p53 by C47 and C61 at the indicated concentrations was assessed in HCT116 WTp53 cells by Western blotting. **D)** Readthrough induction of an EGFP-R213X-FLAG construct by C47 and C61 as assessed in the H1299EGFP- X-FLAG reporter cells. GAPDH was used as loading control.

To further examine the capacity of C47 and C61 to induce readthrough of nonsense mutant *TP53*, H1299p53R213X cells carrying transfected full-length R213X nonsense mutant *TP53* were treated with C47 at 5 µM, 10 µM, 20 µM and 40 µM and C61 at 5 µM and 10 µM (Figure 2A), using G418 at 25 µM as positive control. p53 was detected by antibody DO−1. The results show a dose-dependent increase of full-length p53 protein by both compounds. To confirm these results, H1299p53R213X-FLAG cells carrying full-length R213X *TP53* cDNA with a C-terminal FLAG tag were treated with C47 at 5 µM, 10 µM, 15 µM, 20 µM and C61 at 10 µM and 20 µM (Figure 2B). Full-length p53 was detected by FLAG antibody. This revealed a similar induction of full-length p53 as shown in Figure 2A.

It is conceivable that spontaneous readthrough may produce detectable amounts of full-length protein, especially if expression of the nonsense mutant gene is driven by a strong promoter. Indeed, low levels of full-length p53 were detected even in the untreated H1299p53R213X and H1299p53R213X-FLAG cells (Figure 2A-B). To examine if C47 and C61 could cause stabilization of p53 which could be misinterpreted as induction of translational readthrough, we assessed p53 levels in wild type (WT) *TP53*-carrying HCT116 human colorectal carcinoma cells upon treatment with C47 and C61. The Mdm2 inhibitor Nutlin−3a was used as positive control. As shown in Figure 2C, C47 stabilized WT p53 to some extent but much less potently than Nutlin−3a. C61 did not cause any p53 stabilization at any of the tested concentrations. We also used the reporter cell line H1299EGFP-X-FLAG carrying a construct containing the EGFP coding sequence followed by a *TP53* R213X premature termination codon with flanking sequences (AAC ACC CAA upstream of the TGA stop codon and CTC ACG GTG downstream) and a FLAG tag. Western blotting revealed a robust induction of FLAG expression following treatment with either C47 or C61, supporting a genuine translational readthrough effect of both compounds (Figure 2D).

### C47 and C61 combination treatment with G418 and eRf3a degraders

**Figure 3.**
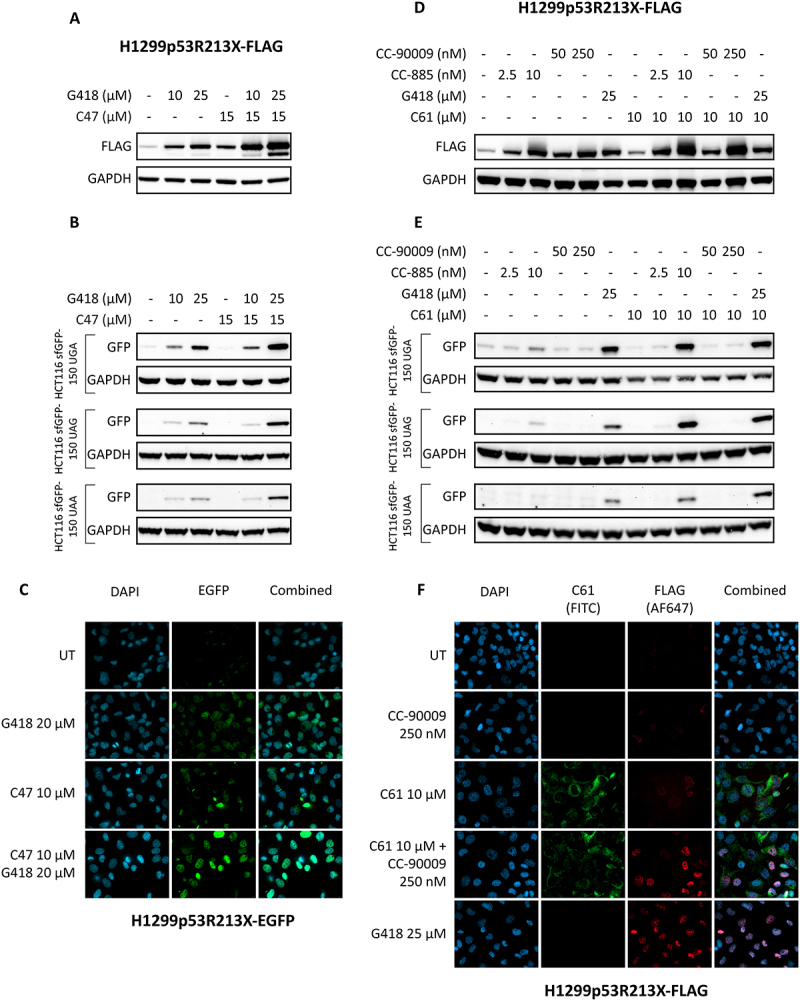
**Combination treatments with C47 and G418 or C61 and CC − 885 or CC − 90009. A, B)** Western blot showing H1299p53R213X-FLAG cells **(A)** or HCT116-sfGFP150 UGA/UAG/UAA cells **(B)** treated with C47 or G418 alone or in combination and blotted for FLAG (full-length p53) or GFP (full-length GFP), respectively. GAPDH was used as loading control. **C)** Immunofluorescence staining of H1299p53R213X-EGFP cells treated with C47 or G418 alone or in combination. **D, E)** Western blot showing H1299p53R213X-FLAG cells **(D)** or HCT116-sfGFP150-UGA/UAG/UAA cells **(E)** treated with CC−885, CC−90009, G418 alone or in combination with C61 or C61 alone and blotted for FLAG (full-length p53) or GFP (full-length GFP), respectively. GAPDH was used as loading control. **F)** Immunofluorescence staining of H1299p53R213X-FLAG cells treated with CC−90009 or C61 alone or in combination. G418 was used as positive control.

C47 and C61 have a relatively modest readthrough effect by themselves. We therefore tested them in combination with other known readthrough-inducing compounds to determine if a more potent readthrough effect could be achieved. Combination treatment with C47 and the aminoglycoside G418 in H1299p53R213X-FLAG cells at the indicated concentrations gave a robust increase in full-length p53 compared to each single treatment alone (Figure 3A). The combination of C47 and G418 was also tested in HCT116 cells transfected with three different stop codon variants of the sfGFP150 gene (UGA, UAG and UAA). G418 alone induced translational readthrough of all sfGFP reporter construct in a dose-dependent manner. No induction of full-length sfGFP by C47 alone at 15 µM was observed at any of the three stop codons. The combination treatment, on the other hand, gave a robust increase in full-length sfGFP protein levels (Figure 3B). The combination treatments were also tested for p53 stabilization in HCT116sfGFP150-UGA cells, which carry WT *TP53*. The results showed no increase in p53 levels at any of the tested concentrations **(Suppl. Fig. S3A)**. We also examined the effect of combination treatment with C47 and G418 in H1299p53R213X-EGFP cells in which translational readthrough could be visualized by EGFP fluorescence. This confirmed that the combination of C47 and G418 resulted in a marked increase in readthrough induction compared to each single treatment alone (Figure 3C). However, the combination treatment with C61 and G418 did not increase readthrough to any major extent according to Western blotting (Figure 3D).

A recent study identified the eRF degraders CC−885 and CC−90009 as potent readthrough inducers [37]. We first tested whether C47 and/or C61 had any effect on eRFs in H2199-p53R213X-FLAG cells. Protein levels of FLAG, eRF1 and eRF3a were analysed by Western blotting upon treatment with C47 or C61. Single treatment with C47 did not affect levels of eRF3a but reduced eRF1 expression **(Suppl. Fig. S3B)**, while C61 had no effect on either eRF3a or eRF1 **(Suppl. Fig. S3C)**. We also tested C47 and C61 in combination with CC−885 and CC−90009 in H1299p53R213X-FLAG cells to determine if any of the drug combinations would have a synergistic effect. No evidence for synergistic induction of translational readthrough was detected by Western blotting when comparing treatment with C47, CC−885 or CC−90009 single treatments and their combinations **(Suppl. Fig. S3B)**. However, we observed a robust synergistic readthrough effect after combination of C61 (10 µM) and CC−885 (10 nM) as well as C61 (10 µM) and CC−90009 (250 nM) (Figure 3D). We then proceeded by treating the three HCT116sfGFP150-UGA/UAG/UAA reporter cell lines with the same combinations as shown in Figure 3D. Western blotting revealed a modest induction of full-length sfGFP protein following treatment with CC−885 (10 nM) in the HCT116sfGFP150-UGA and HCT116sfGFP150-UAG cells. No induction of translational readthrough could be detected following treatment with CC−90009 or C61 in any of the three HCT116sfGFP150 reporter cell lines. However, combination treatment with C61 at 10 µM and CC−885 at 10 nM caused a strong synergistic increase in translational readthrough (Figure 3E). WT p53 protein levels were not increased by any of the treatment conditions, indicating that none of these treatment combinations stabilize p53 **(Suppl. Fig. S3A, right panel)**.

We also performed immunofluorescence staining using H1299p53R213X-FLAG cells treated with C61 at 10 µM and CC−90009 at 250 nM alone or in combination, using and G418 at 25 µM as positive control. The results show a weak induction of readthrough by each compound alone, while the combination treatment generated a robust induction of readthrough as assessed by FLAG immunostaining (Figure 3F). Since C61 has strong fluorescence in the green spectrum as shown using the FITC-filter, anti-FLAG antibody conjugated to Alexa fluor 647 was used for readthrough detection to avoid interference by C61 fluorescence. The fluorescence from C61 is visualized more clearly in **Suppl. Fig. S3D** where H1299p53R213X cells seeded onto coverslips were treated with C61 or DMSO as negative control for 48 h before assessing drug accumulation using fluorescence microscopy at 488 nm (without any antibody, dye or reporter construct). Based on this morphological analysis, we propose that C61 accumulates mainly in mitochondria that are visualized as elongated intracellular networks as a result of mitochondrial fusion or small globular structures from mitochondrial fission.

### Combination treatment that produces synergistic readthrough induction

**Figure 4.**
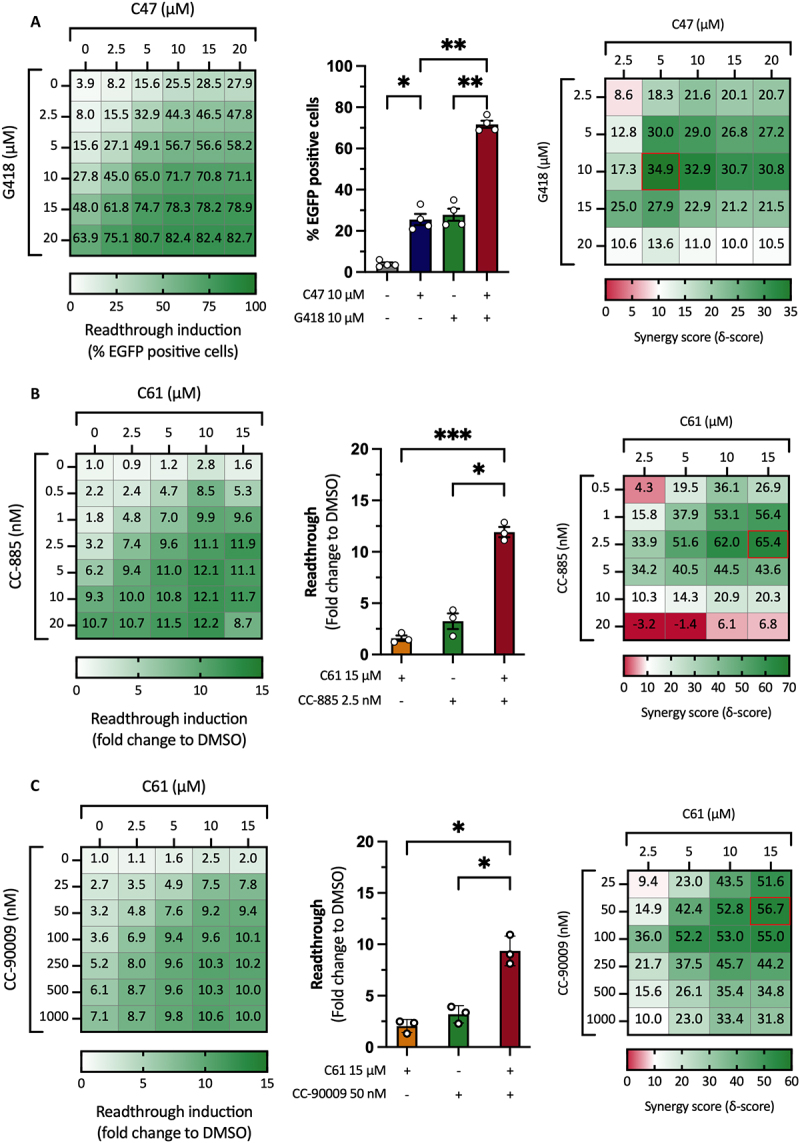
**Combination treatments act synergistically. A)** Left panel: Assessment of readthrough induction by flow cytometry in H1299p53R213X-EGFP cells following treatment with C47 and G418 alone or combination at several concentrations. Middle panel: Selected concentrations presented in a bar chart showing statistically significant increased readthrough upon combination as compared to the single treatments. Right panel: Synergy scores for the different combinations presented as a heatmap. **B, C)** Left panels: Assessment of readthrough induction by ELISA in H1299p53R213X-FLAG cells following treatment with C61 and CC−885 **(B)** or C61 and CC−90009 **(C)** alone or in combination. Middle panels: Selected concentrations of C61 and CC−885 **(B)** or C61 and CC−90009 **(C)** presented in bar charts showing statistically significant increased readthrough upon combination treatment as compared to the single treatments. Right panels: Synergy scores for C61 and CC−885 **(B)** or C61 and CC−90009 **(C)** presented as heatmaps. Repeated measures one-way ANOVA followed by Tukey’s multiple comparisons test was used for statistical analysis (*p ≤ 0.05, **p ≤ 0.01, ***p ≤ 0.001). SynergyFinder 3.0 was used to determine synergy scores using the ZIP model.

In order to verify that the readthrough induction observed following combination treatment with C47 and G418 was synergistic, we assessed readthrough with a more quantitative method. H1299p53R213X-EGFP and H1299p53R213X-FLAG cells were treated with C47 and G418 at 2.5 µM, 5 µM, 10 µM, 15 µM and 20 µM alone or in combination and analysed by FACS. The H1299p53R213X-FLAG cells were used to subtract any minor fluorescence introduced by the compounds. A threshold was set based on the untreated cells for each replicate (*n* = 4), and % EGFP positive cells were measured for all treatments. The full dataset is represented as a heat map (Figure 4A, left panel). C47 at 10 µM and G418 at 10 µM alone or in combination are also shown in a bar chart showing a statistically significant increase in % EGFP positive cells following combination treatment compared to the individual treatments (Figure 4A, middle panel). In **Suppl. Fig. S4A**, synergy calculations for all combinations are demonstrated in its most simplistic form. A higher value for the combination treatment compared to the sum of the two individual treatments found in Figure 4A is indicative of synergy. For instance, C47 at 10 µM resulted in 25.5% EGFP positive cells and G418 at 10 µM resulted in 27.8% EGFP positive cells; thus, the calculated synergy threshold for this combination is 53.3% (25.5 + 27.8 = 53.3). The measured value for the combination treatment was 71.7%, indicating synergy (71.7–53.3 = 18.4). We also analysed the FACS data using the online tool SynergyFinder 3.0 [36]. The overall ZIP synergy score was 21.89 ± 1.95 with peak ZIP ∂-scores at 34.9, indicating strong synergy at several concentrations. The ∂-scores are shown as a heatmap (Figure 4A, right panel).

To avoid complications arising from the strong fluorescence of C61, a non-fluorescence strategy had to be used for the C61 synergy experiments. We opted for ELISA using H1299p53R213X-FLAG cells treated with C61 and CC−885 (Figure 4B) or C61 and CC−90009 (Figure 4C) alone or in combination. Readthrough induction for all treatments was normalized to untreated cells for each replicate (*n* = 3). The results for C61 and CC−885 are depicted as a heat map showing fold change over DMSO (Figure 4B, left panel). One selected concentration is represented in a bar chart showing a statistically significant increase of the combination treatment compared to the two single treatments (Figure 4B, middle panel). Again, synergy calculations for all C61 and CC−885 combinations are shown in a heatmap where a higher value for the combination as compared to the sum of two individual treatments indicates synergy (e.g. C61 15 µM 1.6 + CC−885 2.5 nM 3.2 = synergy threshold 4.8; the measured value was 11.9-fold change over DMSO for the combination treatment) **(Suppl. Fig. S4B)**. In order to make the dataset fit the Synergyfinder tool equations, the raw data had to be normalized to range between 0 and 100. Therefore, the highest readthrough induction in each replicate was set to ‘100% effect’ and all other samples were normalized to the highest value. These data were then analysed using SynergyFinder. This gave an overall ZIP synergy score of 30.14 ± 4.6 and peak ZIP ∂-score values at around 65, indicative of very strong synergy. The ∂-scores are summarized in a heatmap (Figure 4B, right panel).

The C61 and CC−90009 combination was analysed as described for the C61 and CC−885 combination, showing a maximum fold increase over untreated cells of around 10 (Figure 4C, left panel). Selected concentrations presented as a bar chart showed a statistically significant increase of the combination treatment compared to the single treatments (Figure 4C, middle panel). The higher value of the combination treatment as compared to the sum of the individual treatments confirmed a robust synergy for several concentrations (e.g. C61 15 µM 2.0 + CC−90009 50 nM 3.2 = synergy threshold 5.2; the measured value was 9.4-fold change to DMSO for the combination treatment) **(Suppl. Fig. S4C)**. The overall ZIP synergy score for the C61 and CC−90009 combination was 35.56 ± 4.07, reaching peak ZIP ∂-scores at around 57, indicative of strong synergy between C61 and CC−90009 (Figure 4C, right panel).

### C47 induces readthrough of nonsense mutant PTEN and synergizes with G418

**Figure 5.**
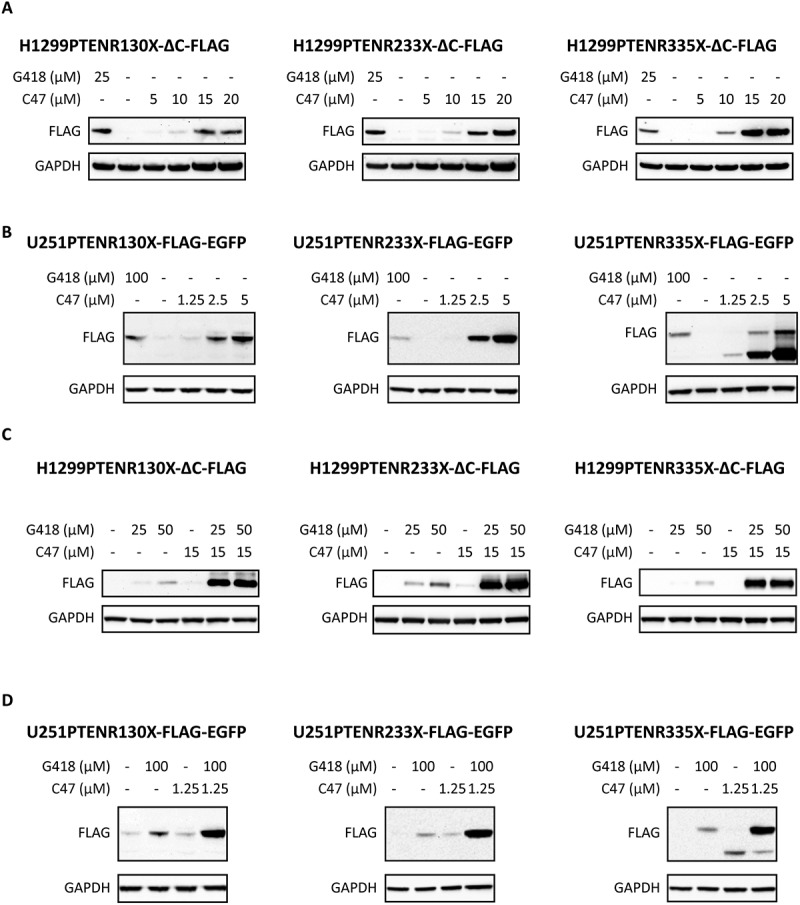
**Readthrough induction of nonsense mutant PTEN by C47 and combination treatment with G418. (A, B)** Western blot analysis of C47 or G418 single treatments in H1299PTENR130X-ΔC-FLAG, H1299PTENR233X-ΔC-FLAG or H1299PTENR335X-ΔC-FLAG cells **(A)** or U251PTENR130X-FLAG-EGFP, U251PTENR233X-FLAG-EGFP or U251PTENR335X-FLAG-EGFP cells **(B)**. **C, D)** Western blot analysis of C47 and G418 combination treatment in H1299PTENR130X-ΔC-FLAG, H1299PTENR233X-ΔC-FLAG or H1299PTENR335X-ΔC-FLAG cells **(C)** or U251PTENR130X-FLAG-EGFP, U251PTENR233X-FLAG-EGFP or U251PTENR335X-FLAG-EGFP cells **(D)**. Readthrough was detected with the FLAG antibody in all Western blots. GAPDH was used as loading control.

To further investigate the ability of C47 and C61 to induce translational readthrough and determine if they could exert a readthrough activity on another tumour suppressor gene, C47 and C61 were tested in H1299 cells stably transfected with the three nonsense mutant *PTEN* constructs (R130X, R233X and R335X) with C-terminal deletion following the stop codon and a C-terminal FLAG tag. C61 did not induce any detectable readthrough in these cells and was therefore not investigated further **(Suppl. Fig. S5A)**. C47 caused a robust induction of full-length PTEN protein in the three H1299 lines with different *PTEN* nonsense mutations and was more potent than the positive control G418 in the R233X and R335X cells (Figure 5A). We also examined readthrough induction by C47 in the *PTEN* null glioblastoma cell line U251 transduced with lentiviral constructs containing the same three *PTEN* nonsense mutants followed by a C-terminal FLAG tag as well as a self-cleaving EGFP reporter. Once again, C47 showed strong readthrough activity in all three *PTEN* nonsense variants (Figure 5B). In U251PTENR335X-FLAG-EGFP cells, C47 induced an additional strong protein band with lower apparent molecular weight than full-length PTEN. This shorter protein detected by FLAG antibody was induced in a dose-dependent manner, like the full-length PTEN protein.

We then treated the three H1299PTEN-R130X/R233X/R335X-∆C-FLAG cell lines with C47 at 15 µM and G418 at 25 µM and 50 µM alone or in combination to determine if the synergistic effect observed for nonsense mutant *TP53* would apply to nonsense mutant *PTEN* as well. The combination of C47 and G418 resulted in a potent induction of readthrough as compared to the single treatments in all the different nonsense variants (Figure 5C). We also tested C47 and G418 combination treatment in the U251PTEN-R130X-FLAG-EGFP, U251PTEN-R233X-FLAG-EGFP and U251PTEN-R335X-FLAG-EGFP cell lines. In concordance with our previous results for both the *TP53* R213X nonsense mutant and the three *PTEN* nonsense mutants in H1299 cells, the combination treatment resulted in a robust and synergistic induction of translational readthrough compared to the single treatments in all the three different U251 *PTEN* nonsense mutant cell lines (Figure 5D).

### Combination treatment with C47 and G418 induces PTEN-dependent growth inhibition in U251 cells

**Figure 6.**
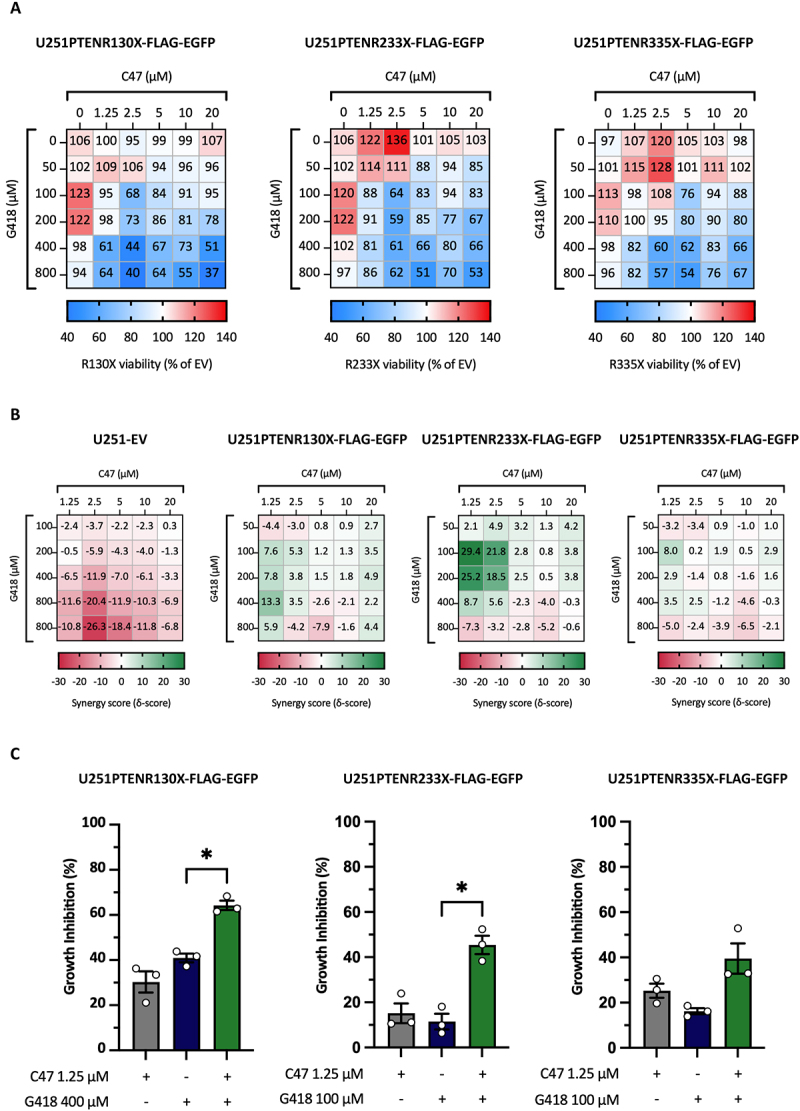
**Biological effect of combination treatment with C47 and G418 in U251 nonsense mutant PTEN cells. A)** WST−1 data showing difference in cell viability between U251 nonsense mutant PTEN sublines and empty vector cells. Values for nonsense mutant PTEN cells are shown as percentage of empty vector cell viability. **B)** Synergy scores presented as heatmaps for all U251 sublines. **C)** the most synergistic concentrations of C47 and G418 presented in bar charts. A statistically significant increase in growth inhibition upon combination treatment as compared to the single treatments is indicated for the PTEN R130X and R233X cells. Repeated measures one-way ANOVA followed by Tukey’s multiple comparisons test was used for statistical analysis (*p ≤ 0.05, **p ≤ 0.01, ***p ≤ 0.001). SynergyFinder 3.0 was used to determine synergy scores using the ZIP model.

To study the biological implications of the substantial induction of full-length PTEN protein following combination treatment with C47 and G418 (Figure 5D), we performed WST−1 cell viability assay. Dose–response curves from single treatment with C47 or G418 revealed no PTEN-dependent effect on cell viability in any of the three U251 nonsense mutant PTEN cell lines **(Suppl. Fig. S6A)**. We then assessed the biological response following C47 and G418 combination treatment. Figure 6A shows difference in cell viability between the three U251 nonsense mutant PTEN cell lines and the empty vector control cells as heatmaps. Results are presented as percentage cell viability compared to the cell viability of the empty vector cells for each treatment combination. The nonsense mutant PTEN cell lines showed reduced cell viability compared to the empty vector control cells at several concentrations of C47 and G418, indicating a PTEN-dependent effect.

To investigate if combination treatment with C47 and G418 can induce a synergistic biological response in the U251 sublines, the WST−1 data were analysed using the SynergyFinder analysis tool and presented as heatmaps (Figure 6B). We observed the strongest synergy in the U251R233X cells, reaching a peak ZIP ∂-score at 29.4. A more moderate synergy was detected in the U251R130X subline with a peak ZIP ∂-score of 13.3, and only additive effects were seen in the U251R335X subline. Interestingly, combination treatment with C47 and G418 was not synergistic in the U251 empty vector control cell line but rather antagonistic, indicating that the observed synergy between C47 and G418 is PTEN-dependent. The most synergistic C47 and G418 treatment combinations for each U251 subline are presented as bar charts in Figure 6C. Statistical significance was reached for the C47 and G418 combination compared with G418 alone in the U251R130X and U251R233X cell lines.

Taken together, our results demonstrate that the novel candidate readthrough-inducing compounds C47 and C61 can induce moderate levels of full-length p53 and/or PTEN protein as single treatments. However, they synergize with the aminoglycoside G418 (C47) or eukaryotic release factor degraders CC−885 and CC−90009 (C61) to induce substantially higher levels of translational readthrough. The combination of C47 and G418 has a synergistic suppressive effect on cell viability in the nonsense mutant PTEN cells.

## Discussion

Nonsense mutations in tumour suppressor genes such as *TP53*, *PTEN* and *APC* are common in a wide range of tumour types [15]. Pharmacological induction of translational readthrough in tumours carrying such nonsense mutations is an attractive therapeutic approach. Tumour cells with a nonsense mutant tumour suppressor gene still carry the full coding sequence of the gene. In theory, all that is needed for reactivation of WT function is to unleash the nonsense mutant gene from its restraints by translational readthrough. Yet there are currently no approved drugs targeting nonsense mutations in *TP53, PTEN* or other tumour suppressor genes.

In this study, we performed high-throughput screening of more than 30 000 compounds in HDQ-P1 human breast cancer cells harbouring homozygous R213X nonsense mutant *TP53* [32] and identified C47 and C61 as novel candidate readthrough-inducing compounds. As readout, we assessed p53 protein expression by immunofluorescence staining with monoclonal antibody DO−1. Since this antibody detects an N-terminal p53 epitope [38], it does not distinguish full-length p53 from C-terminally truncated p53. Immunostaining with an antibody that recognizes a C-terminal p53 epitope would have facilitated the screening, but available C-terminal p53 antibodies PAb421 [38] gave a too low signal to noise ratio with our immunostaining protocol. Therefore, validation of hits by Western and ELISA was crucial in order to confirm induction of full-length p53 protein.

Since spontaneous readthrough may lead to expression of low levels of full-length protein despite the presence of a premature termination codon [39], increased levels of full-length protein could be due to protein stabilization rather than true translational readthrough. This is particularly relevant for p53 that is normally regulated at the level of protein stability. We found that C61 did not stabilize full-length p53 in WT *TP53* HCT116 cells, whereas C47 had a weak stabilizing effect. Moreover, both compounds induced the FLAG epitope in reporter cells carrying EGFP-R213X-FLAG, and C47 was able to induce full-length PTEN in cells carrying nonsense mutant *PTEN*, thus showing potent activity in a non-p53 context. Taken together, these observations support a genuine translational readthrough activity. Nonetheless, we can at present not exclude that the observed increase in full-length p53 is due, at least in part, to protein stabilization. Formal proof for translational readthrough can only be obtained by ribosome profiling in order to assess whether the increased levels of full-length p53 protein are associated with an increased ribosome occupancy 3’ of the R213X premature termination codon in *TP53* mRNA, as we have previously demonstrated for 5-Fluorouridine [31].

Neither C47 nor C61 was able to induce detectable levels of translational readthrough as single treatments in the HCT116sfGFP150-UGA/UAG/UAA reporter cells, which could reflect poor readthrough activity. However, it is possible that the sequence context in these cells affects the readthrough process, as the nucleotides surrounding the stop codon are known to impact the termination efficiency [26]. Especially the + 4 nucleotide (the nucleotide directly downstream of the stop codon) greatly influences the stringency in translation termination (G > A > U > C, where G gives the highest termination efficiency) [40,41]. In the three HCT116sfGFP150-UGA/UAG/UAA reporter cells, the + 4 nucleotide is a G, which should result in a robust termination of translation (see Table 2 for a full list of the nucleotide context surrounding the stop codons of all constructs used). In the H1299EGFP-X-FLAG cells, the UGA stop codon is followed by the + 4 nucleotide C, resulting in a relatively weak termination signal and optimal readthrough conditions. This could account for the different efficiencies in readthrough induction by C47 and C61 alone between these readthrough reporter cell lines.

**Table 2.** **Nucleotide sequences surrounding the premature termination codon.** The nucleotide context before and after the premature termination codon for each construct is used in this study. The premature termination codon is shown in bold.

Cell line	Stop codon nucleotide context
H1299PTENR130X-∆C-FLAG	GGA AAG GGA **TGA** ACT GGT GTA
H1299PTENR233X-∆C-FLAG	GGA CCC ACA **TGA** CGG GAA GAC
H1299PTENR335X-∆C-FLAG	AAA GCC AAC **TGA** TAC TTT TCT
H1299EGFP-X-FLAG	AAC ACC CAA **TGA** CTC ACG GTG
H1299p53-R213X	AAC ACT TTT **TGA** CAT AGT GTG
H1299p53-R213X-FLAG	AAC ACT TTT **TGA** CAT AGT GTG
H1299p53-R213X-∆C-FLAG	AAC ACT TTT **TGA** CAT AGT GTG
H1299p53-R213X-EGFP	AAC ACT TTT **TGA** CAT AGT GTG
HCT116sfGFP150-TGA	AAC AGC CAC **TGA** GTG TAC ATC
HCT116sfGFP150-TAG	AAC AGC CAC **TAG** GTG TAC ATC
HCT116sfGFP150-TAA	AAC AGC CAC **TAA** GTG TAC ATC
U251PTENR130X-FLAG-EGFP	GGA AAG GGA **TGA** ACT GGT GTA
U251PTENR233X-FLAG-EGFP	GGA CCC ACA **TGA** CGG GAA GAC
U251PTENR335X-FLAG-EGFP	AAA GCC AAC **TGA** TAC TTT TCT

Previous reports by us and others have shown that a combination therapy approach can be beneficial in terms of translational readthrough output. In these studies, G418 was tested in combination with compounds acting on either eRF3 (CC−885 & CC−90009) [37] to prevent translation termination or MDM2 (Nutlin−3a) [22] to prevent p53 protein degradation following induction of translational readthrough. These studies demonstrated that compounds that act on different targets involved in protein translation or protein turnover can act synergistically to potentiate the net translational readthrough output, thus achieving substantially higher levels of full-length protein. This principle was adopted in this current study in which novel readthrough inducing compounds C47 and C61 with unknown mechanisms of action were investigated in combination with CC−885, CC−90009 or G418 to evaluate potential synergistic effects.

C47, but not C61, was able to induce PTEN∆C-FLAG or full-length PTEN-FLAG protein in H1299 and U251 cells with three different nonsense *PTEN* mutations (R130X, R233X and the R335X), again arguing that C47 possesses true translational readthrough activity. Moreover, we found that C47 and G418 combination treatment had a striking readthrough effect in all three *PTEN* nonsense mutants. The combination treatment resulted in a massive induction of translational readthrough as compared to the single treatment in both the lung adenocarcinoma (H1299) and glioblastoma (U251) cell backgrounds. C47 also induced a protein with lower apparent molecular weight than full-length PTEN in the U251PTENR335X-FLAG-EGFP cells. It is unclear if this protein is an N-terminally truncated variant of full-length PTEN detected by the FLAG antibody against the C-terminal FLAG-tag. An alternative shorter PTEN protein with a molecular weight corresponding roughly to the observed lower protein band could in theory be generated by translation initiation from a downstream in-frame methionine (Met35). Further investigation of the origin of this C47-induced lower molecular weight protein is clearly required.

One limitation of this study is that the mechanisms of action of C47 and C61 remain poorly understood. However, our data allow some tentative conclusions. C47 does not affect eRF3a levels but does cause a marked reduction in eRF1 levels **(Suppl. Fig. S3B**). eRF1 and eRF3a are dependent on each other to form a complex to initiate the translation termination process [17]. eRF1 binds to the decoding centre of the ribosome and eRF3 is a GTPase that catalyzes the peptide release. This suggests that C47, CC−885 and CC−90009 act on the same pathway to induce translational readthrough and could also explain the lack of synergy between the compounds. Liquid chromatography-mass spectrometry (LC-MS) revealed that neither C47 nor C61 bind RNA (data not shown). Taken together, our data suggest a link between the ability of C47 to inhibit eRF1 and its readthrough activity. This should be properly evaluated in future studies to elucidate the exact mechanism of action. C61, on the other hand, did not affect eRF1 or eRF3a, indicating an alternative and unknown mechanism. The only conclusion that can be drawn at present is that the mechanism of action of C61 most probably differs from that of G418 (that binds ribosomal RNA) and the eRF3a degraders.

Our analysis of p53 downstream target activation following treatment with C61 and C47 alone or in combination with G418, CC−885 or CC−90009, did not reveal any increased expression of any of the downstream targets. Nuclear/cytosolic fractionation of H1299p53R213X-FLAG cells treated with C61 and CC−885 showed that p53 induced by the treatment was able to enter the nucleus (data not shown). This was confirmed by p53 immunostaining upon treatment with C47 or C61 alone or in combination with G418 or CC−90009. It is conceivable that readthrough induced by C47 or C61 results in the incorporation of an incorrect amino acid at the R213X premature termination codon, which could cause p53 misfolding and failure to bind DNA and transactivate target genes. In contrast, G418 is known to mainly induce incorporation of arginine at the UGA stop codon which would result in translation of WT p53 [42]. Another possibility is that truncated p53, which is induced more potently by both C47 and C61 than G418 (Figure 2A), somehow inhibits the transcriptional transactivation activity of full-length p53. Expression of misfolded mutant p53 or N-terminally truncated p53 isoforms are known to abrogate WT p53 function in cancer cells by a dominant negative mechanism and/or by amyloid formation [43,44]. It is possible that high levels of C-terminally truncated p53 may have a similar impact on WT p53 function. Due to our ambiguous results regarding p53 downstream target activation following translational readthrough, we focused our analysis of biological effects of combination treatment with C47 and G418 on the nonsense mutant PTEN cell lines U251R130X/R233X/R335X.

In conclusion, we have identified two novel compounds with potential readthrough activity in nonsense mutant *TP53* and/or *PTEN*. Their mechanisms of action are yet to be determined and further research is needed to fully understand what cellular processes are affected. The ability of C47 and C61 to synergize with G418 or CC−885 and CC−90009 may have interesting implications for future development of cancer therapy targeting tumours with nonsense mutation in *TP53* or *PTEN.*

## Supplementary Material

Supplemental Material

## Data Availability

The data that support the findings of this study are available from the corresponding author KGW upon request: https://doi.org/10.5281/zenodo.761493.
