# Supplementary material for: Novel compounds that synergize with aminoglycoside G418 or eRF3 degraders for translational readthrough of nonsense mutant TP53 and PTEN
Source: RNA Biol. 2023 Jun 20;20(1):368–83. doi: 10.1080/15476286.2023.2222250 (PMC10283442; doi:10.1080/15476286.2023.2222250)

Figure 1A H1299 p53R213XΔC-FLAG

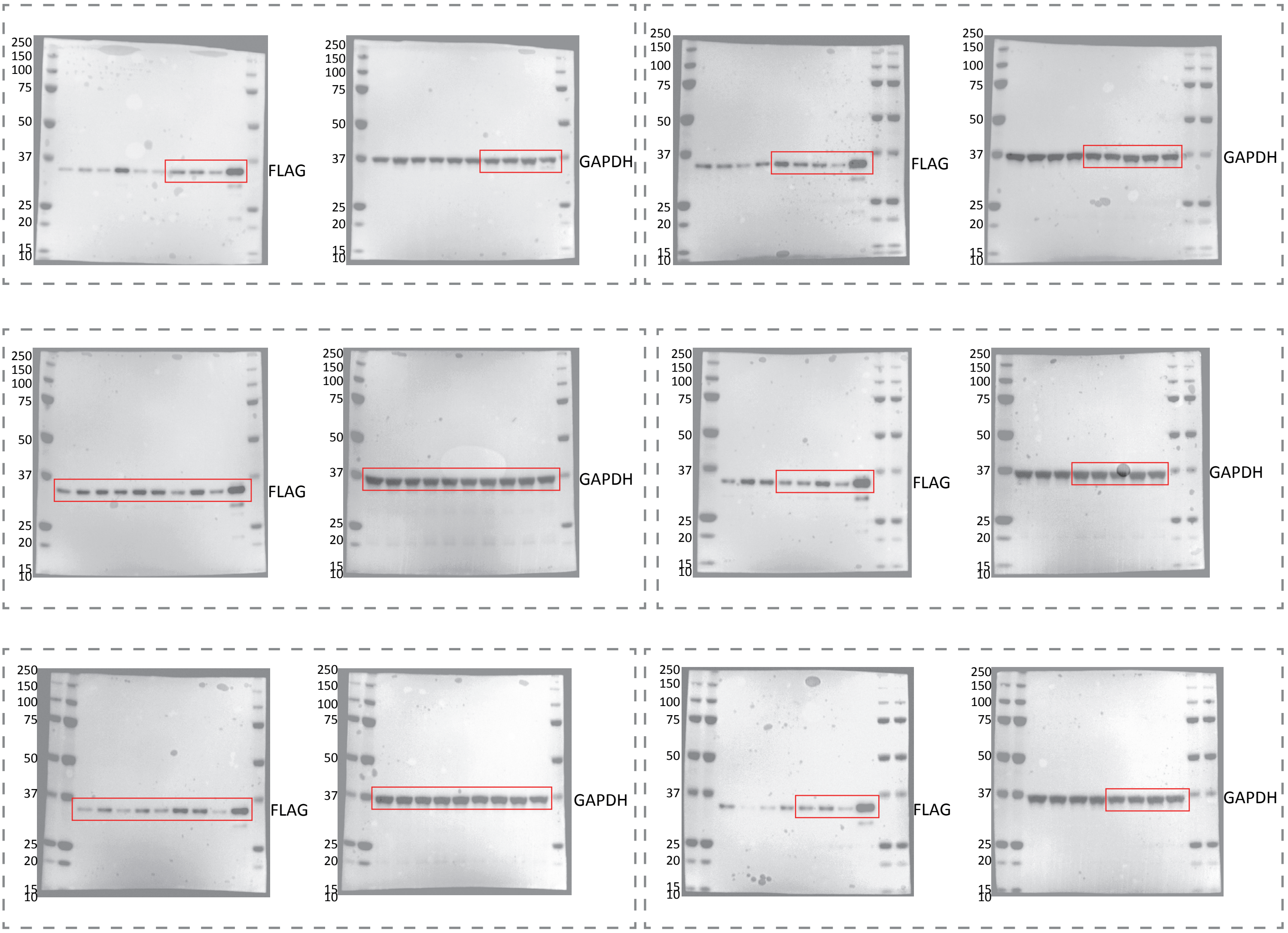

Figure 2A, LEFT H1299 p53R213X

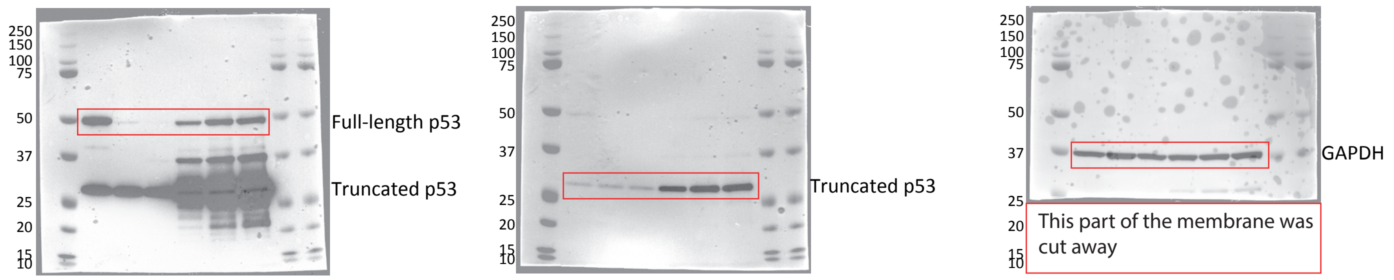

Figure 2B, LEFT H1299 p53R213X-FLAG

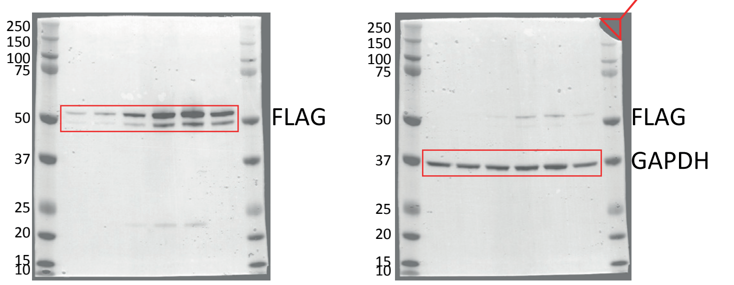

Figure 2C, LEFT HCT116 WT p53

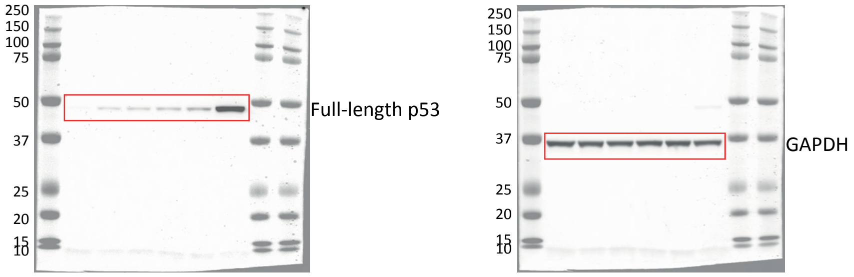

Figure 2D, LEFT H1299 EXF

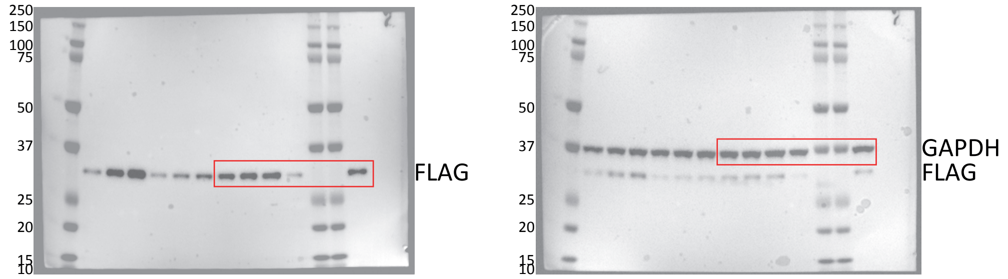

Figure 2A, RIGHT H1299R213X

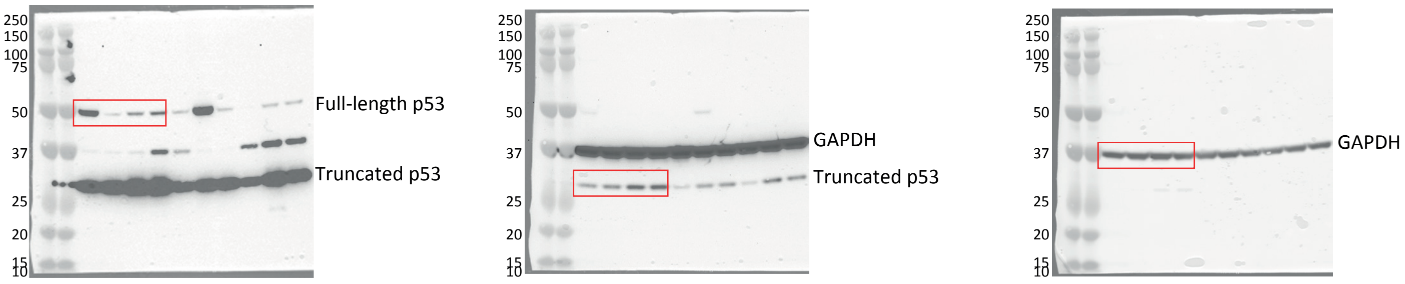

Figure 2B, RIGHT H1299R213X-FLAG

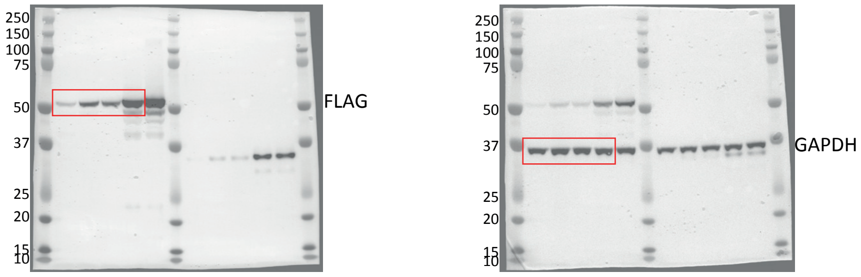

Figure 2C, RIGHT HCT116 WT p53

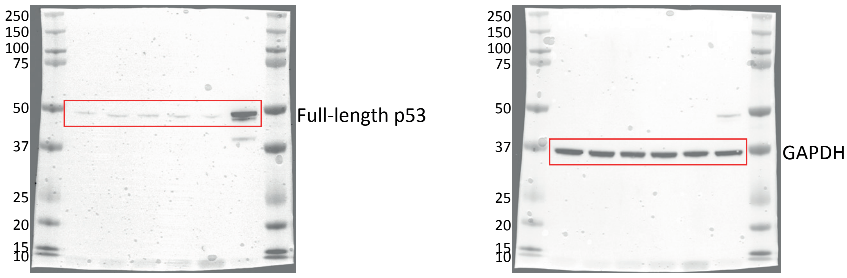

Figure 2D, RIGHT H1299 EXF

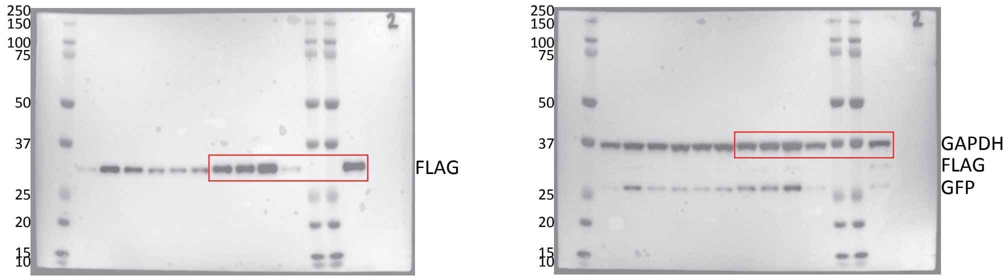

Figure 3A H1299 p53R213X-FLAG

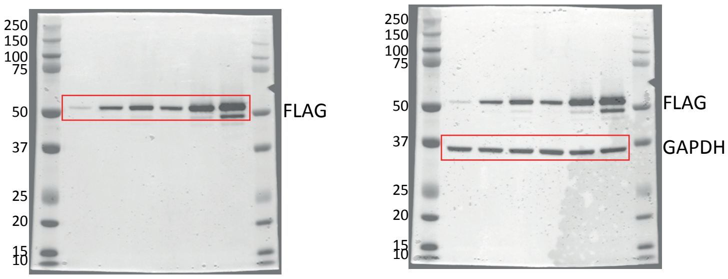

Figure 3B HCT116 sfGFP150UGA

Figure 3B HCT116 sfGFP150UAG

Figure 3B HCT116 sfGFP150UAA

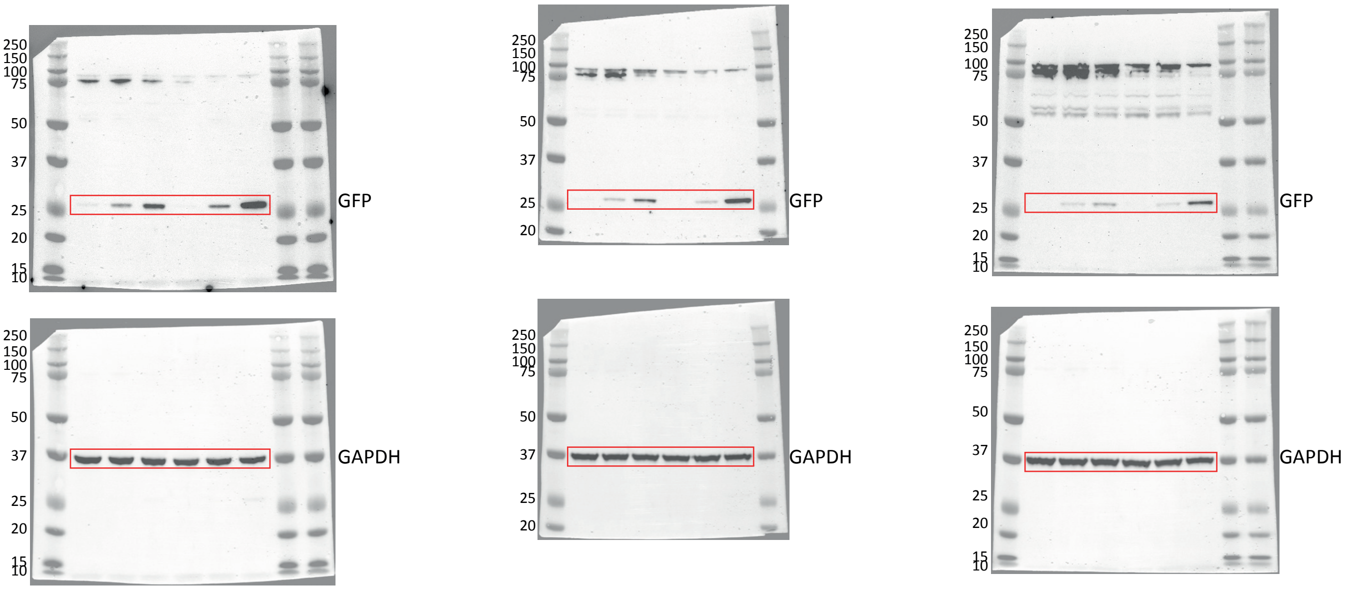

Figure 3D H1299 p53R213X-FLAG

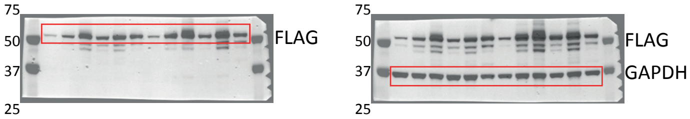

Figure 3E HCT116 sfGFP150UGA

Figure 3E HCT116 sfGFP150UAG

Figure 3E HCT116 sfGFP150UAA

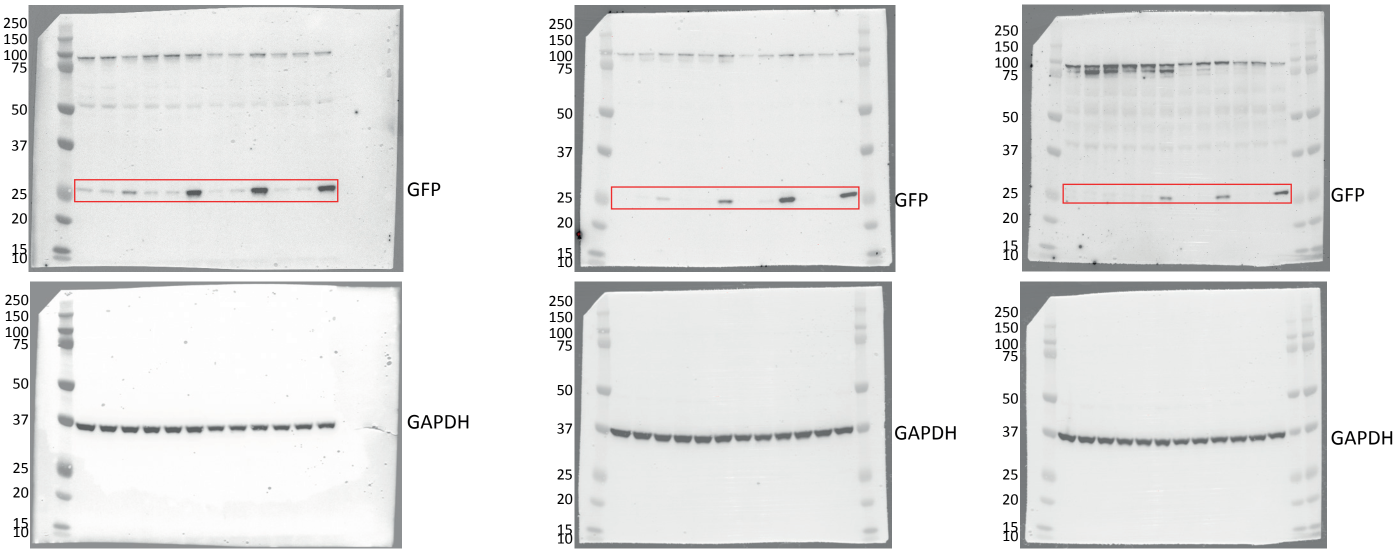

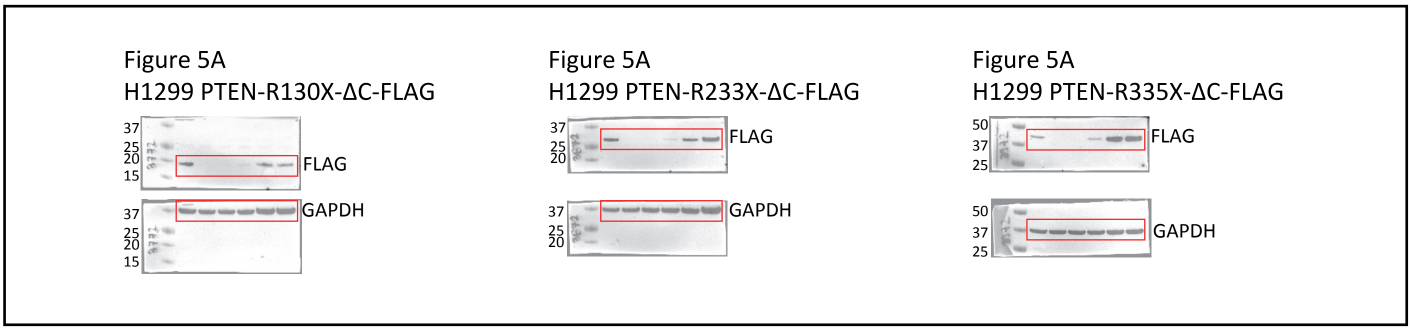
Figure 5A
H1299 PTEN-R130X-ΔC-FLAG
37
25
20
15
FLAG
37
25
20
15
GAPDH
Figure 5A
H1299 PTEN-R233X-ΔC-FLAG
37
25
20
FLAG
37
25
20
GAPDH
Figure 5A
H1299 PTEN-R335X-ΔC-FLAG
50
37
25
FLAG
50
37
25
GAPDH

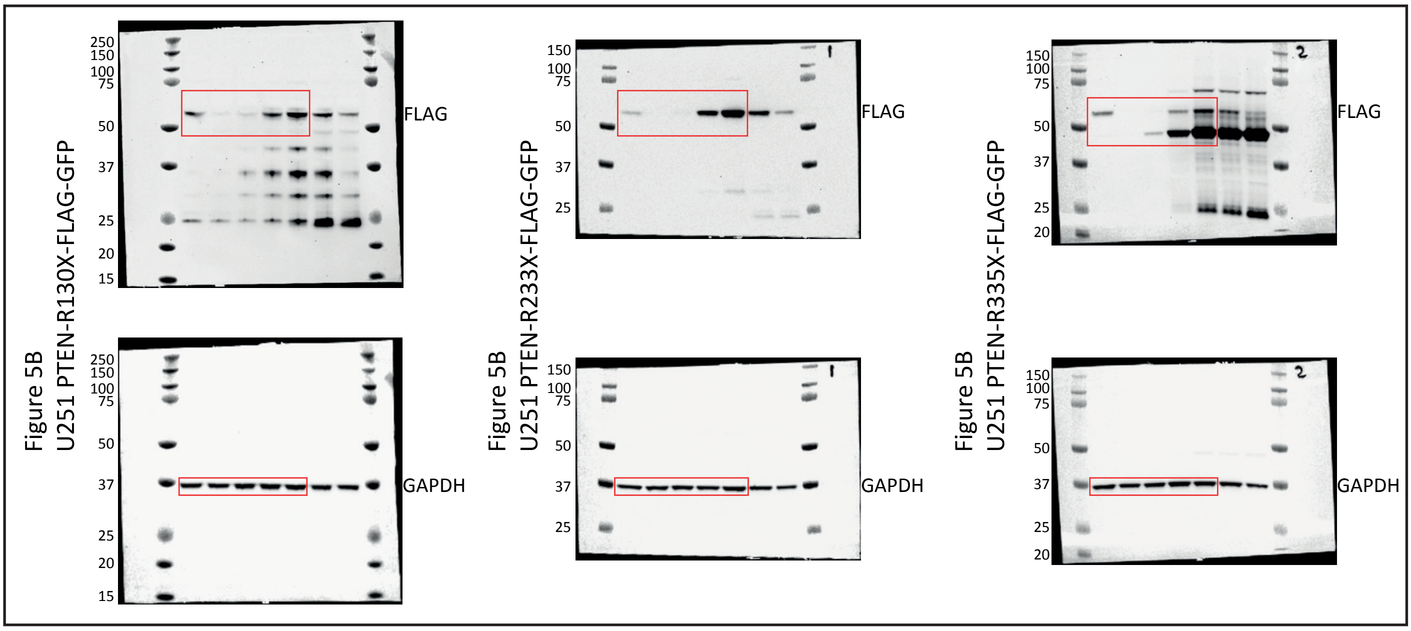
Figure 5B
U251 PTEN-R130X-FLAG-GFP
250
150
100
75
50
37
25
20
15
FLAG
250
150
100
75
50
37
25
20
15
GAPDH
Figure 5B
U251 PTEN-R233X-FLAG-GFP
150
100
75
50
37
25
FLAG
150
100
75
50
37
25
GAPDH
Figure 5B
U251 PTEN-R335X-FLAG-GFP
150
100
75
50
37
25
20
FLAG
150
100
75
50
37
25
20
GAPDH

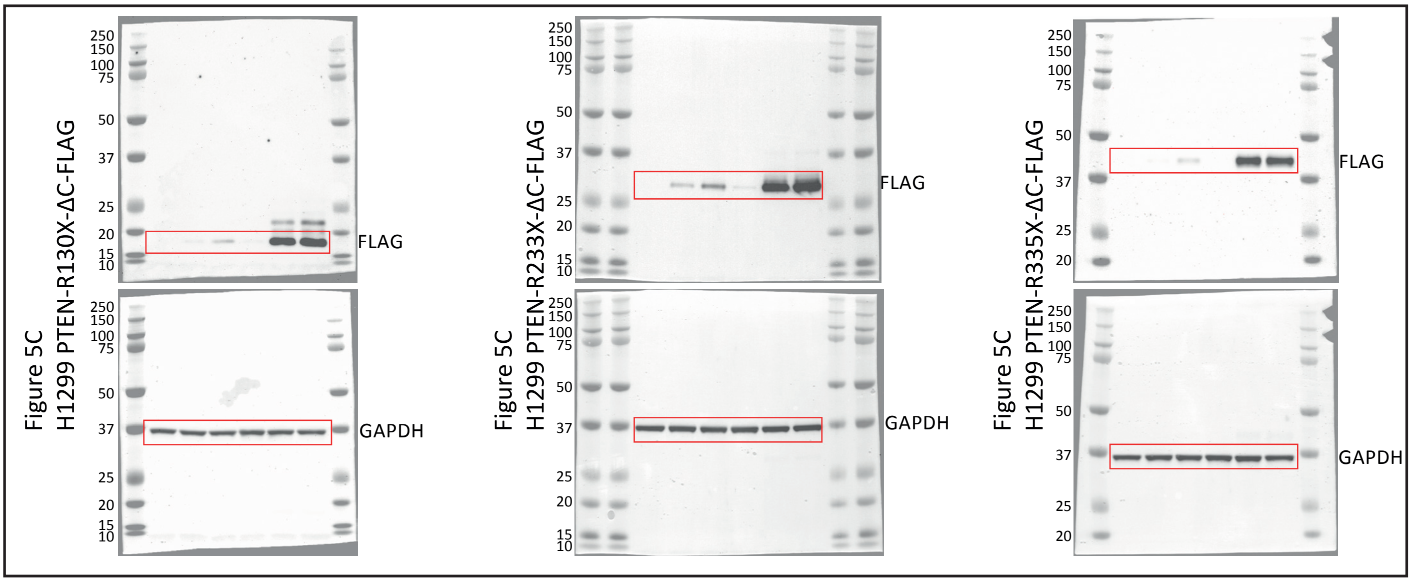
Figure 5C
H1299 PTEN-R130X-ΔC-FLAG
250
150
100
75
50
37
25
20
15
10
FLAG
250
150
100
75
50
37
25
20
15
10
GAPDH
Figure 5C
H1299 PTEN-R233X-ΔC-FLAG
250
150
100
75
50
37
25
20
15
10
FLAG
250
150
100
75
50
37
25
20
15
10
GAPDH
Figure 5C
H1299 PTEN-R335X-ΔC-FLAG
250
150
100
75
50
37
25
20
FLAG
250
150
100
75
50
37
25
20
GAPDH

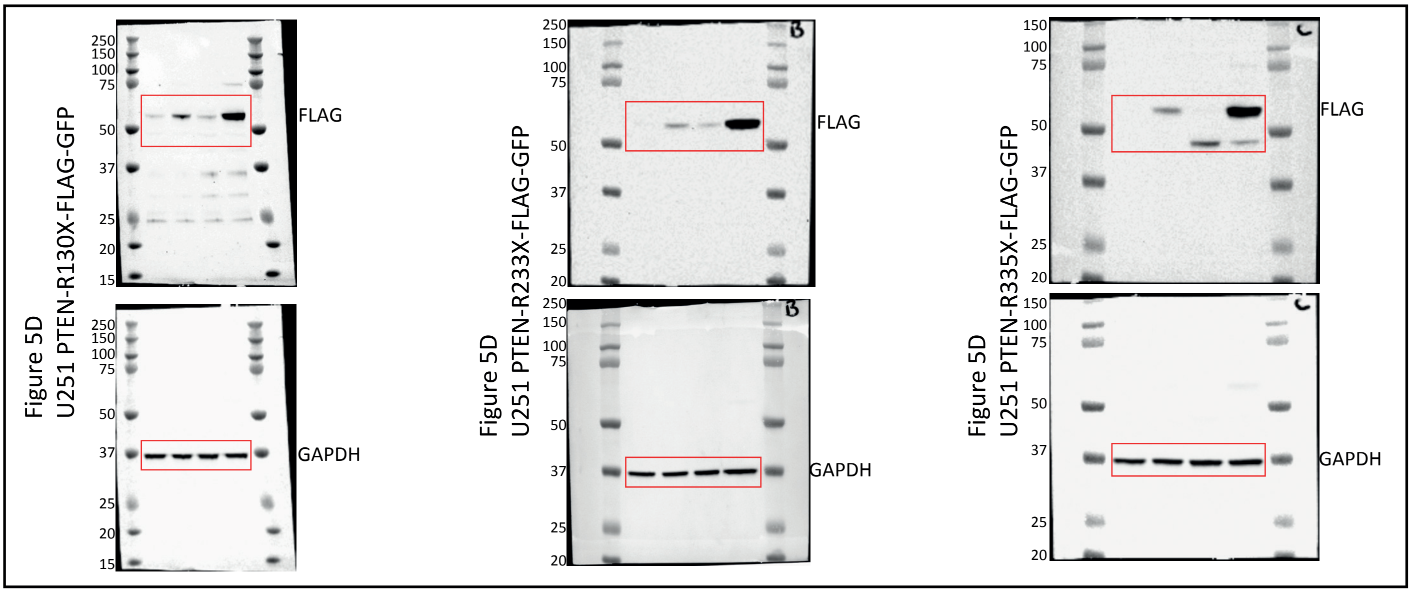
Figure 5D
U251 PTEN-R130X-FLAG-GFP
250
150
100
75
50
37
25
20
15
FLAG
250
150
100
75
50
37
25
20
15
GAPDH
Figure 5D
U251 PTEN-R233X-FLAG-GFP
250
150
100
75
50
37
25
20
FLAG
250
150
100
75
50
37
25
20
GAPDH
Figure 5D
U251 PTEN-R335X-FLAG-GFP
150
100
75
50
37
25
20
FLAG
150
100
75
50
37
25
20
GAPDH

Supplementary Figure 3A
HCT116sfGFP150 UGA C47

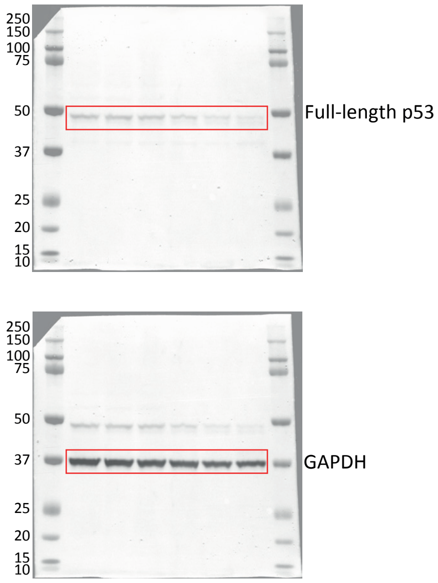

Supplementary Figure 3A
HCT116sfGFP150 UGA C61

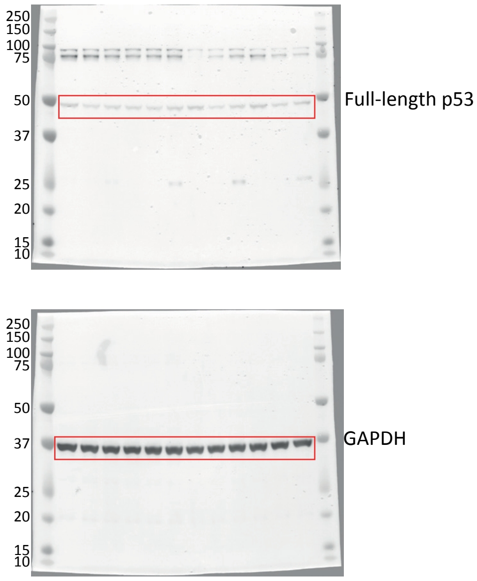

Supplementary Figure 3B H1299p53R213X-FLAG

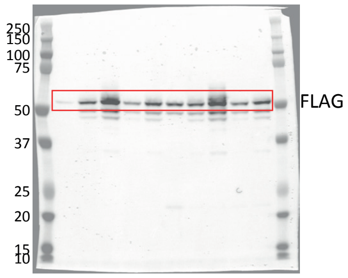

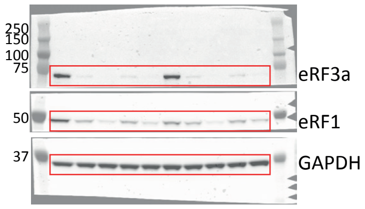

Membrane was first blotted for FLAG, then stripped, cut and reblotted for eRF3a, eRF1 and GAPDH

Supplementary Figure 3C H1299p53R213X-FLAG

Gel 1

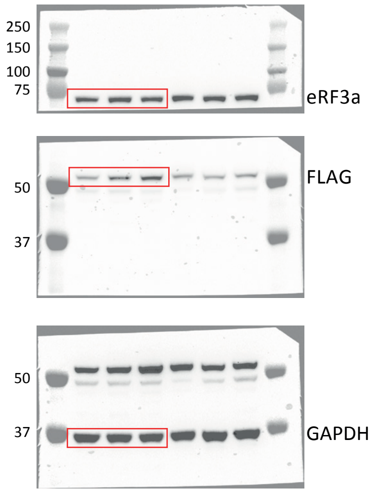

Gel 2

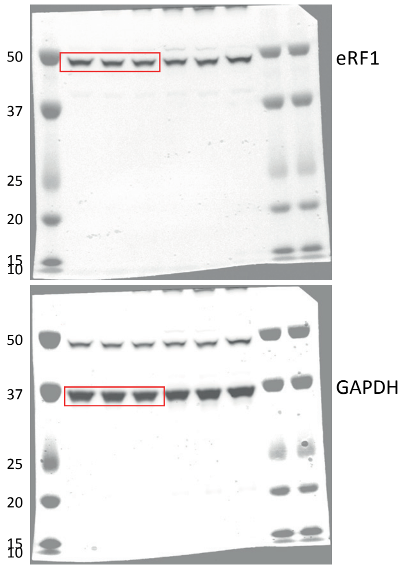

Supplementary Figure 5A
H1299 PTEN-R130X-ΔC-FLAG

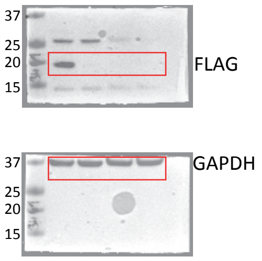

Supplementary Figure 5A
H1299 PTEN-R233X-ΔC-FLAG

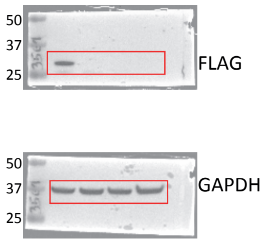

Supplementary Figure 5A
H1299 PTEN-R335X-ΔC-FLAG

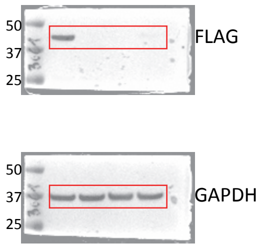

Supplement: Supplemental Material [file KRNB_A_2222250_SM1970.zip › Appendix 1 Uncropped western blots with ladders.pdf]
