# Supplementary figures and images for: Novel compounds that synergize with aminoglycoside G418 or eRF3 degraders for translational readthrough of nonsense mutant TP53 and PTEN
Source: RNA Biol. 2023 Jun 20;20(1):368–83. doi: 10.1080/15476286.2023.2222250 (PMC10283442; doi:10.1080/15476286.2023.2222250)

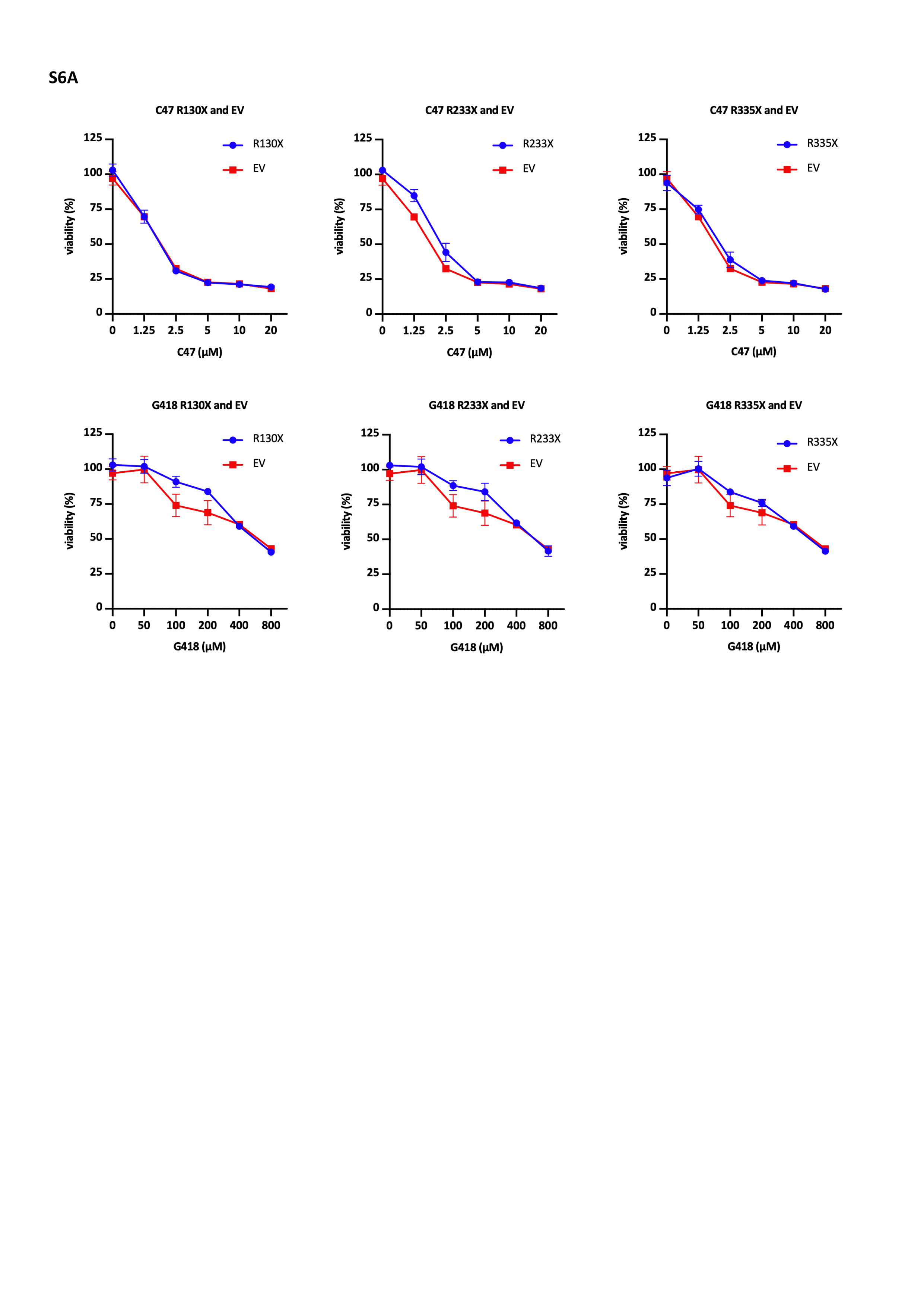

Supplement: Supplemental Material [file KRNB_A_2222250_SM1970.zip › 230529 Main Figures REVISED_Sup. Figure 6.tif]

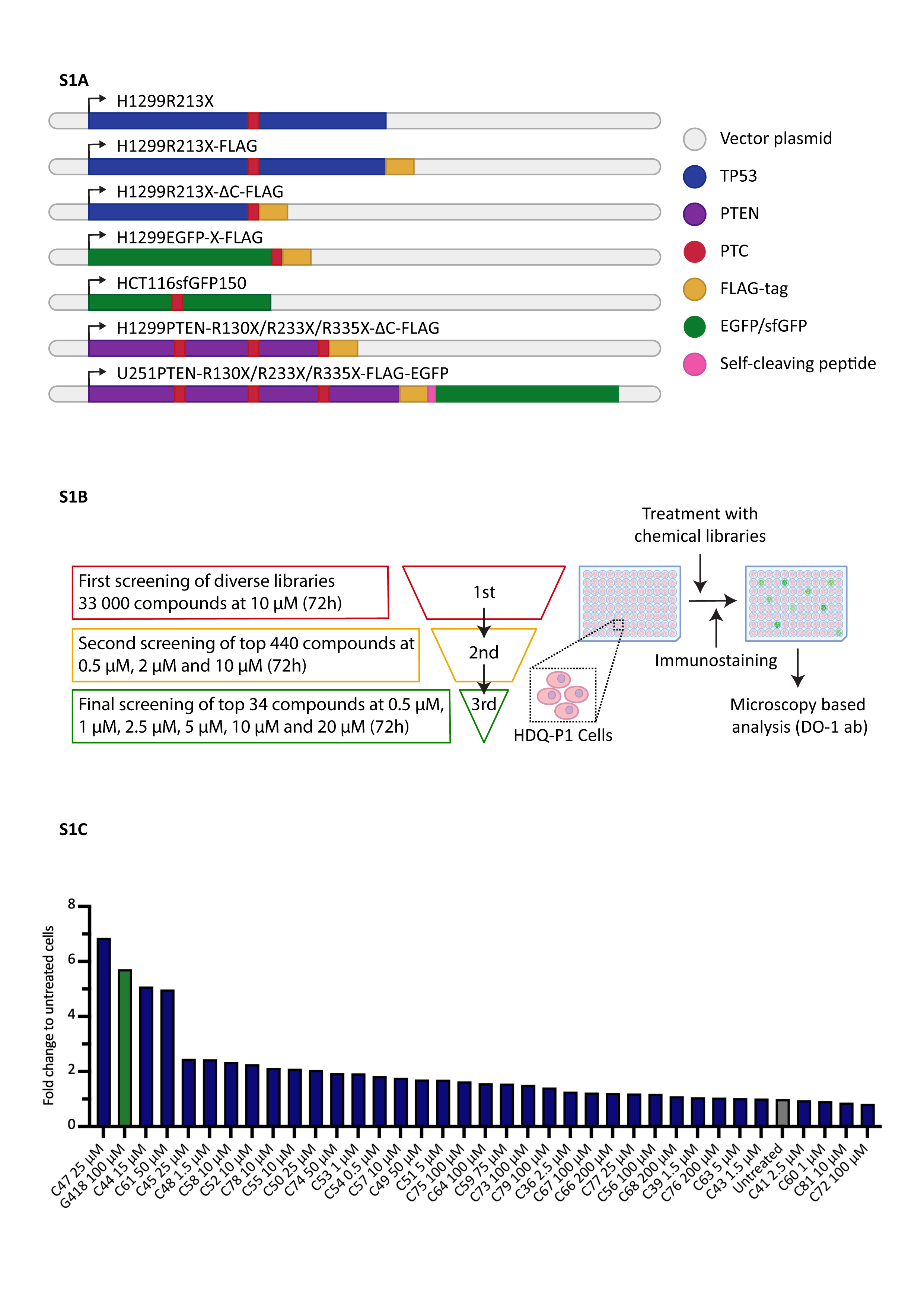

Supplement: Supplemental Material [file KRNB_A_2222250_SM1970.zip › 230529 Main Figures REVISED_Sup.Figure 1.tif]

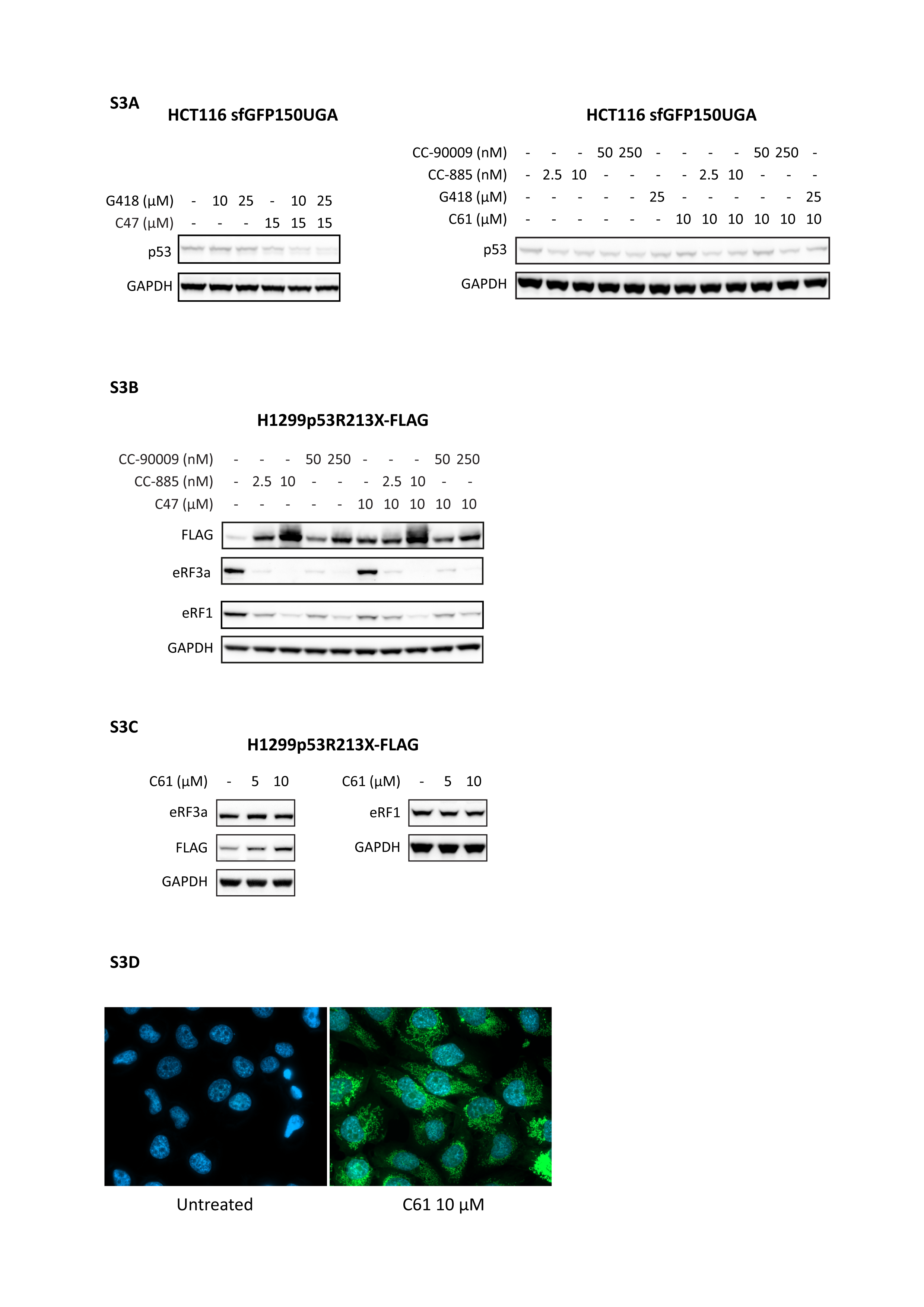

Supplement: Supplemental Material [file KRNB_A_2222250_SM1970.zip › 230529 Main Figures REVISED_Sup.Figure 3.tif]

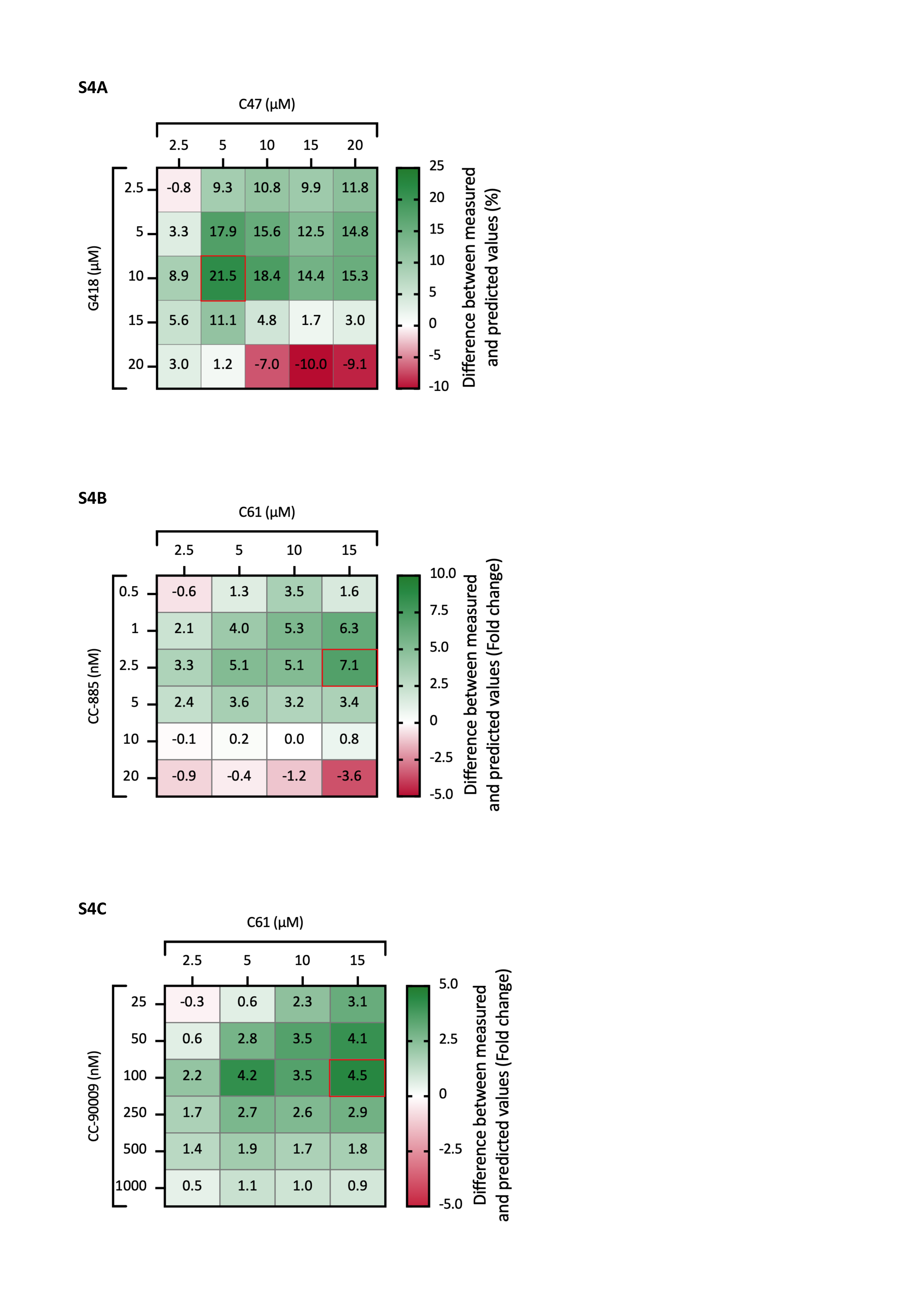

Supplement: Supplemental Material [file KRNB_A_2222250_SM1970.zip › 230529 Main Figures REVISED_Sup.Figure 4.tif]

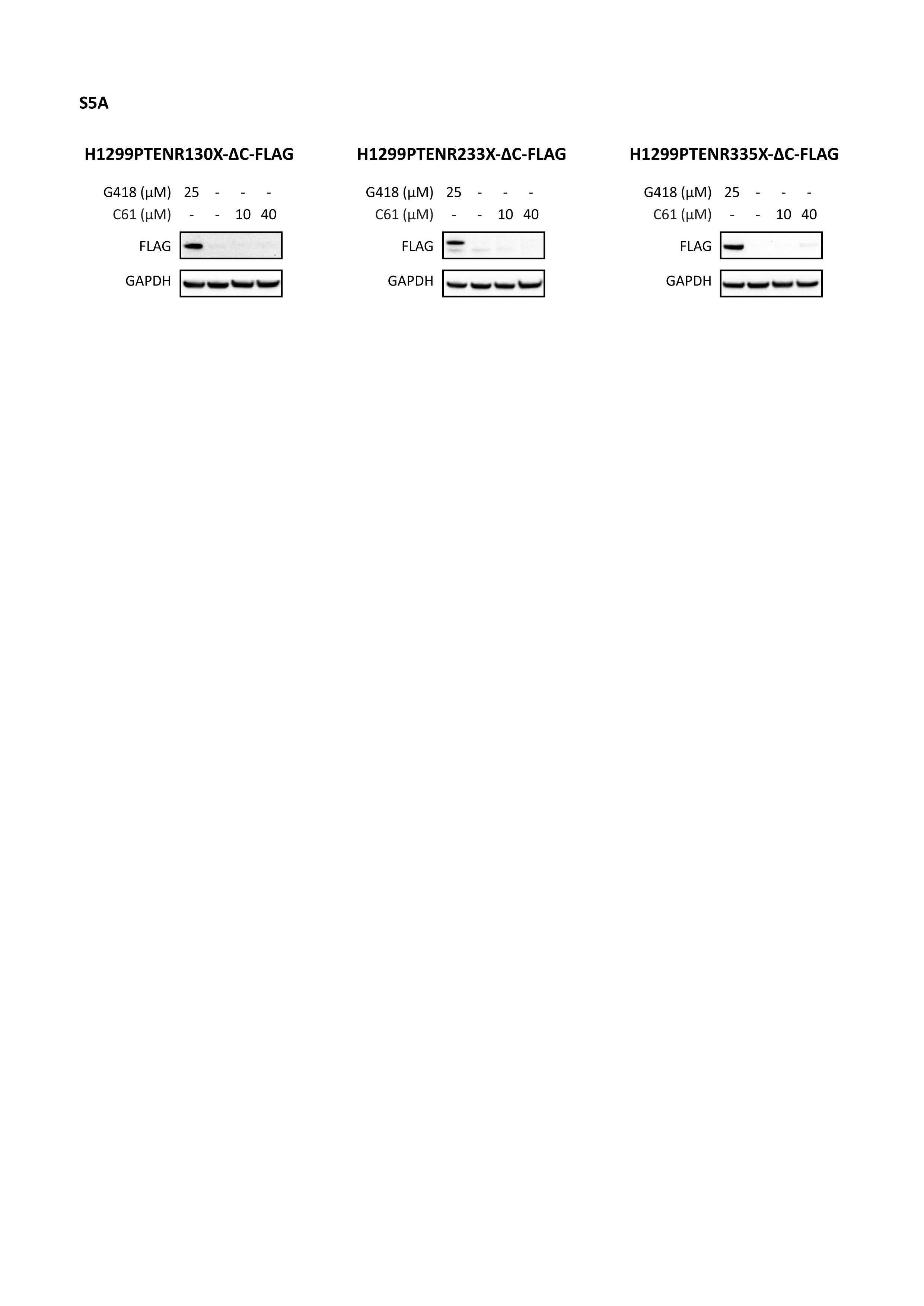

Supplement: Supplemental Material [file KRNB_A_2222250_SM1970.zip › 230529 Main Figures REVISED_Sup.Figure 5.tif]
